# Response of grassland productivity to climate change and anthropogenic activities in arid regions of Central Asia

**DOI:** 10.7717/peerj.9797

**Published:** 2020-08-31

**Authors:** Xu Bi, Bo Li, Lixin Zhang, Bo Nan, Xinshi Zhang, Zihan Yang

**Affiliations:** 1 College of Resources and Environment, Shanxi University of Finance and Economics, Taiyuan, China; 2 School of Natural Resources, Faculty of Geographical Science, Beijing Normal University, Beijing, China; 3 College of Urban and Environmental Science, Peking University, Beijing, China; 4 Institute of Botany, Chinese Academy of Sciences, Beijing, China

**Keywords:** Grassland degradation, Mountain basin system, Climate change, Anthropogenic activities, Net primary productivity (NPP), Quantitative assessment

## Abstract

**Background:**

Quantitative evaluations of the relative impacts of climate change and anthropogenic activity on grasslands are significant for understanding grassland degradation mechanisms and controlling degraded grasslands. However, our knowledge about the effects of anthropogenic activities and climate change on the grassland in a mountain basin system in arid regions of Central Asia is still subject to great uncertainties.

**Methods:**

In this research, we have chosen the net primary productivity (NPP) as an index for revealing grassland dynamics processes. Moreover, the human appropriation of net primary production (NPP_H_), which was calculated as the potential NPP (NPP_P_) minus the actual NPP (NPP_A_), was applied to distinguish the relative influences of climate change and human activities on the grassland NPP variations in a mountain basin system of Central Asia from 2001–2015.

**Results:**

The results indicated that the grassland NPP_A_ showed an increasing trend (35.88%) that was smaller than the decreasing trend (64.12%). The respective contributions of human activity, climate change and the two together to the increase in the NPP_A_ were 6.19%, 81.30% and 12.51%, respectively. Human activity was largely responsible for the decrease in the grassland NPP_A_, with the area experiencing human-induced decreases accounting for 98.21% of the total decreased area, which mainly occurred during spring/autumn pasture and winter pasture. Furthermore, the average grazing pressure index (GPI) values of summer pastures, spring/autumn pasture and winter pastures were 1.04, 3.03 and 1.83, respectively, from 2001–2015. In addition, negative correlations between the NPP and GPI occupied most of the research area (92.41%).

**Discussion:**

Our results indicate that: (i) anthropogenic activities were the primary cause of the reduction in the grassland NPP, especially grazing activities. (ii) For areas where the grassland NPP has increased, precipitation was the dominant climatic factor over temperature in controlling the grassland NPP changes in the study area. (iii) The findings of the current research indicate that some measures should be taken to reduce livestock pressure, and artificial grasslands can be built along the Irtysh River and the Ulungur River to relieve grazing pressure on spring/autumn pastures and winter pastures. Our results could provide reliable information for grassland management and the prevention of grassland degradation in arid regions of Central Asia.

## Introduction

Grasslands are one of the extremely significant terrestrial ecosystems, accounting for almost 30% of the earth’s land area (Houghton, 1994). Additionally, grassland ecosystems are a crucial carbon pool, accounting for about 20% of global soil carbon reserves and playing a significant part in the global carbon cycle (Ahlström et al., 2015). In addition, they link socio-economic development with regional ecological security. The grassland area of China is 3.93 million km^2^, including 866,700 km^2^ of degraded grasslands (Bao et al., 1998). Recent studies have indicated that approximately 90% of the grasslands in northern China have degraded to a certain extent in northern China (Nan, 2005).

Grassland ecosystems are an important component of terrestrial ecosystems. Terrestrial ecosystems have experienced severe changes during the past decades, as climate change and anthropogenic activities together have contributed to dynamic variations in terrestrial ecosystems (Stocker, Plattner & Qin, 2014; Jiang et al., 2015). Currently, people can use remote sensing to monitor terrestrial ecosystem dynamics accurately to a certain extent, but there is no uniform conclusion about the dominant factors and their relative impacts on terrestrial ecosystem changes (Fensholt et al., 2012). Particularly in semiarid and arid areas, climate variation and aggravation due to anthropogenic activities are likely to lead to ecological degradation and severe ecological and financial losses (Wessels, Prince & Reshef, 2008). Therefore, it is of great significance to accurately evaluate and differentiate the individual contributions of anthropogenic activities and climate change to ecosystem changes to formulate effective ecological management and regulation policies.

**Figure 1:**
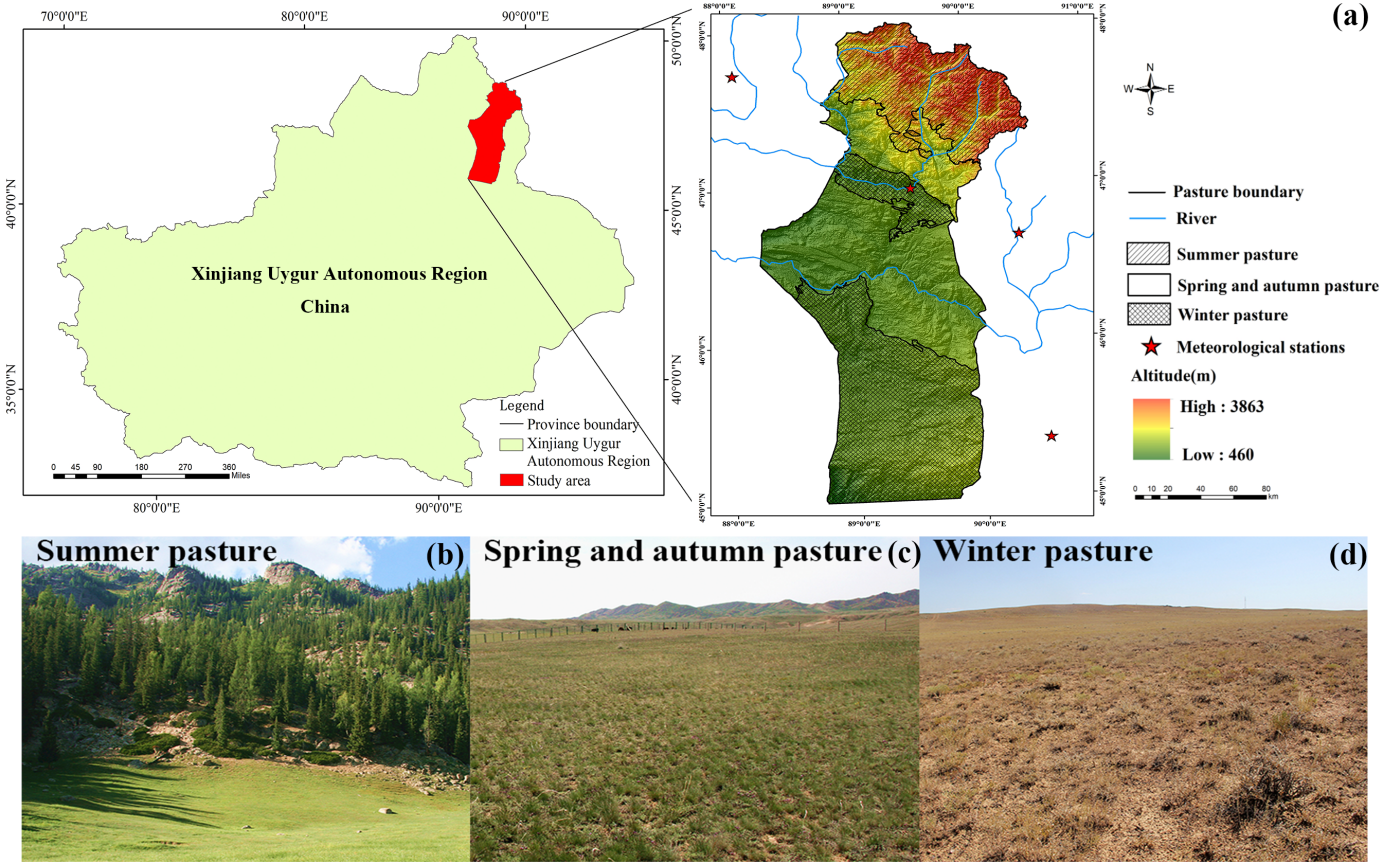
Map of the geographical location of the study area (A); the landscape map of summer pasture (B), spring and autumn pasture (C) and winter pasture (D).

The mountain basin system (MBS) is a typical geomorphic structure in the arid region of central Asia (Zhang, 2001), which is distributed with mountains and basins (Fig. 1). In recent years, the relative impacts of climate change and human activities have been studied on the watershed (Wang et al., 2016a; Wang et al., 2016b; Xu, Wang & Zhang, 2017), regional (Xu, Wang & Zhang, 2016; Liu et al., 2018a; Liu et al., 2018b; Yan et al., 2019; Zheng et al., 2019) and national scales (Thomas et al., 2014; Liu, Li & Li, 2015; Chen et al., 2019). Although there have been studies in Xinjiang (Yang et al., 2017; Zhang et al., 2018), few studies have focused on the MBS. Therefore, our knowledge about the effects of anthropogenic activities and climate change on grassland vegetation variation are still subject to great uncertainties in the MBS in the arid region.

The traditional quantitative evaluation method refers to the development of statistical relationships between driving factors and grassland degradation, such as partial derivatives analysis (Zhang et al., 2016), multiple variable analysis (Li et al., 2018) and principal component analysis (Ma et al., 2007). Nevertheless, these methods are mainly dependent on statistical analysis, ignoring the ecological process of grassland degradation and its driving factors, which can easily lead to greater uncertainty in the results (Becerril-Pina et al., 2015). Additionally, the residual trend (RESTREND) method has been widely carried out by some researchers (Li, Wu & Huang, 2012). However, this method presents certain limitations and uncertainties in differentiating the impacts of human and climatic factors on ecosystems (Chen et al., 2014).

The NPP is the overall quantity of organic matter amassed effectively by vegetation per unit area and time, which not only denote the scope of grassland changes, but also has a substantial impact on the ecosystem carbon cycle (Liang et al., 2015). The NPP is a signal that is responsive to climatic changes and human activities, which can precisely show the reactions of grassland growth to climate and human influences (Potter, Klooster & Genovese, 2012). Therefore, the NPP was adopted as an index with which to disentangle the relative impacts of climate change and anthropogenic activity on ecosystem process changes (Zheng et al., 2006; Wessels, Prince & Reshef, 2008). To better understand the effects of human activities on ecosystems, the anthropogenic appropriation of the net primary production (NPP_H_) was developed by Haberl (1997). Some scholars have applied the NPP_H_ to estimate the effects of anthropogenic activities on ecosystems (Guo et al., 2017; Zhou et al., 2017). This approach provides an effective way to quantitatively analyse the respective influences of anthropogenic activities and climate change on grassland variation.

Fuyun County is a representative MBS in Central Asia, as it is seated in the arid region of Xinjiang, China. Grasslands cover the vast majority of the land in this region; therefore, the grassland ecosystem plays a vital role in social stability and regional ecological security. Due to its poor soil and exiguous vegetation, grassland ecosystems in arid regions are more sensitive to anthropogenic activity and climate change (Seddon et al., 2016). By July 2016, the area of degraded grassland had reached 16,891 km^2^, accounting for 35.06% of the entire grassland area (Fuyun Grassland Ecology Station, 2017). Several studies have indicated that the grasslands of Central Asia have degraded due to frequent anthropogenic activities and climate change (Xi & Sokolik, 2015; Zhang & Ren, 2017). Previous research has reported that the grassland NPP has increased as a result of the increase in precipitation and temperature and the different ecological conservation projects in some areas of China (Zhou et al., 2015). Moreover, some studies have indicated that the grassland NPP has decreased as a result of overgrazing and the arid climate (Wang et al., 2016a; Wang et al., 2016b). Climate change has different impacts on various grassland types in an MBS; therefore, future changes in grassland ecosystems in the future may become more complicated, and we must also consider the intensification of anthropogenic activities. Additionally, the effects of climate change and anthropogenic activities show great spatial heterogeneity among different grassland types in northern Xinjiang (Cudahy et al., 2016). Consequently, local governments cannot easily determine how to manage the grassland in an MBS and identify the areas that have been affected by anthropogenic activity and/or climate change. In addition, grazing activities have shown remarkable effects on grasslands in Fuyun County (Bi et al., 2018), and it is an ideal area in which to study the impacts of climate change and anthropogenic activities on grassland ecosystems.

This research tries to test the following main hypotheses: (1) grassland ecosystem changes present obvious spatial heterogeneity under the background of climate change and anthropogenic activity and (2) the grassland ecosystem variation from 2001 to 2015 was jointly impacted by climate change and anthropogenic activity, but grassland degradation was more related to human activities. Based upon the above hypotheses, the contributions of this research include: (1) exploring the spatial–temporal patterns and dynamics of the grassland NPP in Fuyun County from 2001 to 2015; (2) analysing the relative importance of climate change and anthropogenic activities in grassland changes and (3) studying the major causes of grassland NPP variation in different seasonal pastures. Knowledge of the above results may contribute to grassland resource management and sustainable development and to the prevention of grassland degradation in arid areas with an MBS structure in Central Asia.

These findings will provide a theoretical reference for policy makers aiming to prevent grassland degradation and optimize the management of ecosystems with MBS structures in arid areas, which is critical for regional ecological security and the global carbon cycle. In addition, it will provide reliable information to aid in achieving the sustainable use of grassland resources.

## Materials & Methods

### Study region

**Figure 2:**
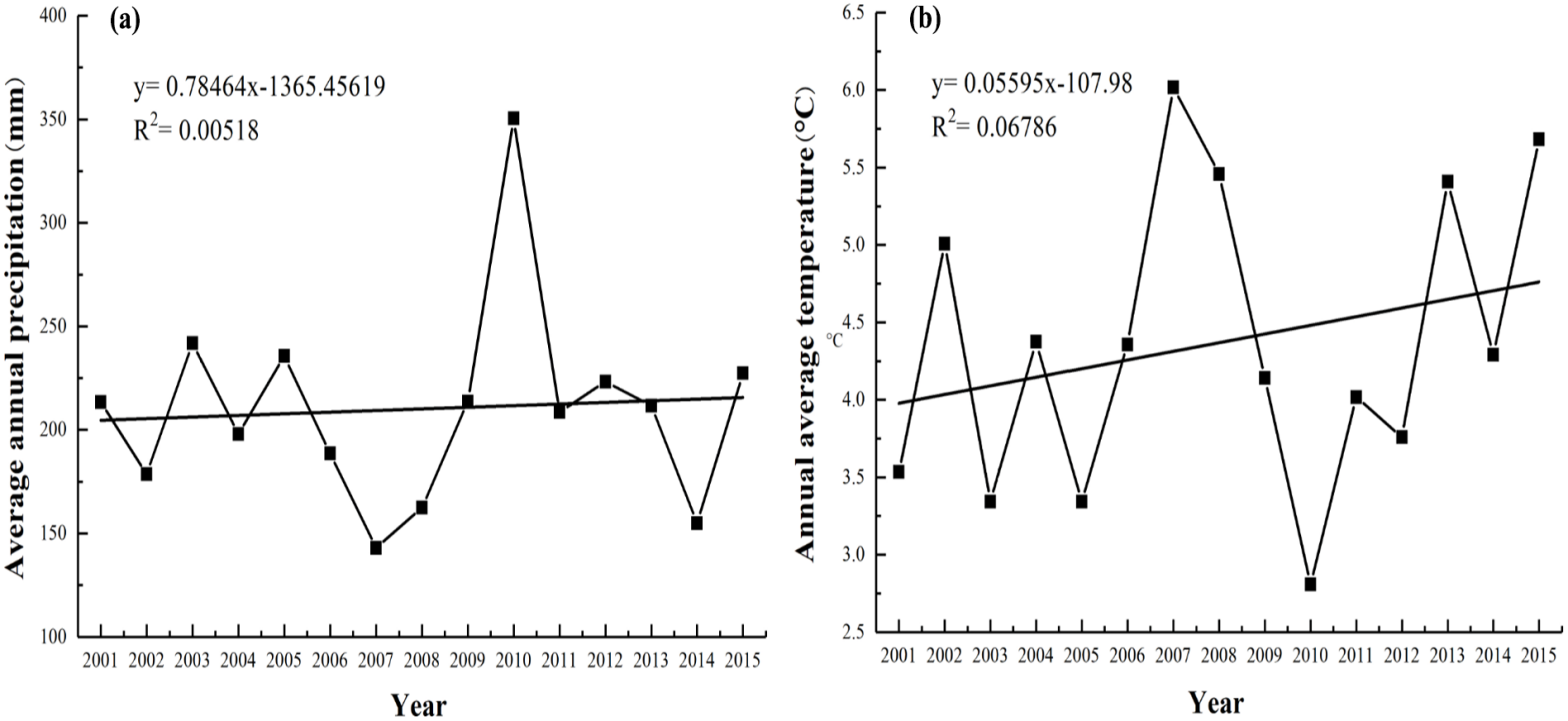
Interannual variation trends of the (A) average annual precipitation(mm) and (B) annual average temperature (°C) from 2001–2015.

The research area is in Fuyun County, China, and covers a latitudinal range of 45°00′–48°03′N and a longitudinal range of 88°10′–90°31′E (Fig. 1A). It lies on the northern border of the Xinjiang Uygur Autonomous Region, stretching from the southern base of the Altai Mountains to the northern region of the Junggar Basin. The research area covers 32,186 km^2^, with altitudes ranging from 317 m to 3,863 m. The study area is located in temperate arid and semi-arid climate zone, with a complex topography that alternates between mountains and basins. The annual average temperature is 4.60 °C (2007–2016), and the average annual precipitation is 208.41 mm (2007–2016) (Fig. 2). The highest value of average annual precipitation occurred in 2010 (350.4 mm), with the lowest in 2007 (143 mm). The highest annual average temperature was in 2007 (6.02 °C), and the lowest was in 2010 (2.81 °C). Grassland is the major type of ecosystem in this area, covering an area of 26,656.33 km^2^, which is 82.82% of the total area. According to the classification system of grassland resources in China, ten types of grasslands are found in Fuyun County. Nomads and livestock migrate among different grasslands at different altitudes depending on the season, and based on the period during which the nomads utilize the grassland, the grassland is divided into various seasonal pastures (summer pasture, spring and autumn pastures, and winter pastures) (Figs. 1B–1D).

### Data sources

#### NDVI (Normalised Difference Vegetation Index) data

In the Carnegie–Ames–Stanford approach (CASA) model (Potter, Klooster & Genovese, 2012) for computing the NPP in Fuyun County, the normalised difference vegetation index (NDVI) data, land cover data, precipitation, temperature and solar radiation were selected as input arguments. NDVI data with a 250 m resolution (MOD13Q1) from 2001 to 2015 were procured from the NASA Land Processes Distributed Active Archive Center (LP DAAC) (https://lpdaac.usgs.gov/). To eliminate the effects of clouds, sandstorms and haze, we adopt the maximum value composite method to synthesize monthly NDVI time series data from 16-day NDVI data. We projected remote-sensing data onto a Lambert conformal conic projection and WGS-84 datum using ArcGIS V10.2.

### Meteorological data

The meteorological data from stations within the Xinjiang Uygur Autonomous Region were obtained from the National Meteorological Information Center (http://data.cma.cn/). The monthly average temperature was collected by 56 meteorological stations, and the total precipitation was recorded by 11 stations within the Xinjiang Uygur Autonomous Region. The solar radiation at other meteorological stations in Xinjiang was calculated based on the 11 sites with solar radiation records (Liu & Li, 1997). The meteorological data were interpolated by the thin-plate smoothing spline method using ANUSPLIN, taking the altitude as the covariable to produce raster images of the whole Xinjiang region with a 250 m spatial resolution, and then the study area was extracted according to the vector boundary of Fuyun County.

### Land cover data

**Figure 3:**
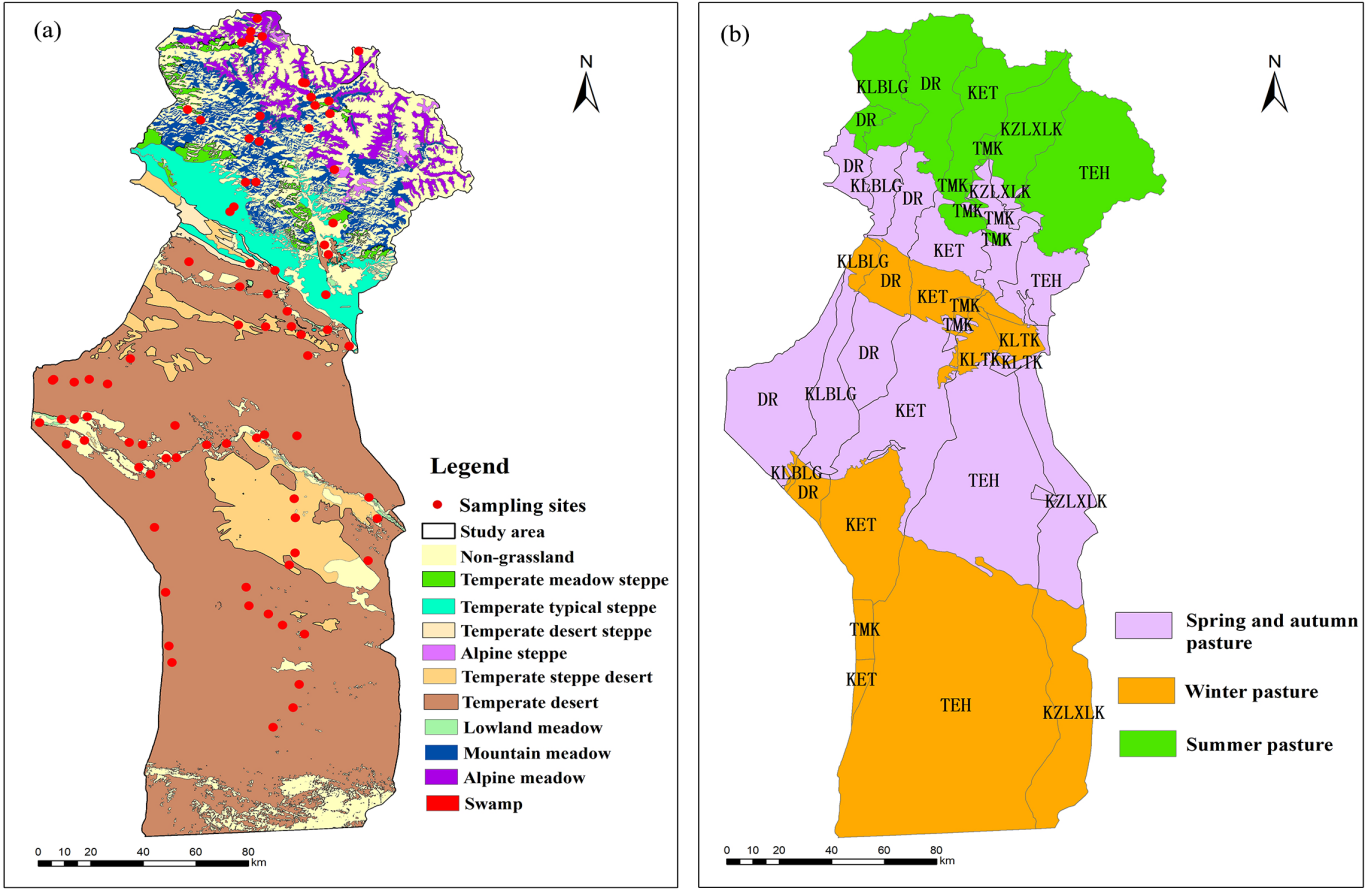
Grassland types in the study area (A) and the regions divided by different townships and seasonal pastures (B). DR, Dure town; KLBLG, Kalabulegen town; KZLXLK, Kezilexilike town; KET, Kuerte town; TMK, Tiemaike town; and TEH, Tuerhong town.

The land cover data were vectorized according to the “Grassland resource type map of Fuyun County” provided by the grassland station of the Xinjiang Uygur Autonomous Region, which was drawn in 2009. In the grassland resource type map of Fuyun County, in addition to grassland, the non-grassland parts include farmland, forests, residential areas, bare land, stone extraction areas, water areas, river beaches and mining areas. The grassland is divided into alpine steppe, alpine meadow, mountain meadow, temperate meadow steppe, temperate typical steppe, temperate steppe desert, temperate desert steppe, lowland meadow, temperate desert, and swamp in light of the classification system of grassland resources in China (Fig. 3A). To ensure the validity and stability of the study, the areas with constant grassland types were selected (Forzieri et al., 2017).

### Biomass data

Biomass data accessible for this study were collected from 83 sampling plots of Fuyun County in August 2015 (Fig. 3A) (Table S1).

### Livestock number data

Livestock number data was obtained from the Twelfth Five-year Plan statistical yearbook of Fuyun County (2016).

## Methods

### Field sampling

There were 83 sampling plots in our study area. The area of each sample plot was 10 m × 10 m, and three quadrats were set in the areas where the distribution of the grassland plant community was relatively uniform. In each quadrat, the above-ground biomass (AGB) portions of the vegetation community and the underground biomass portions of representative species in each grassland type were collected. The biomass was dried at 65 °C for 48 h to obtain a constant mass and then weighed and averaged as the weight of the plots. The amount of dry matter (g m^−2^) converted to carbon (g C m^−2^) needs to be multiplied by the conversion factor. To calculate the actual NPP (NPP_A_), we converted the AGB to the total biomass using the ratio index of each grassland type, and then multiplied the total biomass by 0.475 to convert it to the NPP (g C m^−2^) (Zhou, 1996; Scurlock et al., 1999).

### Quantitative calculations of the NPP

Three types of NPP (NPP_A_, NPP_P_ and NPP_H_) were defined to distinguish grassland variations influenced by anthropogenic activities and climate change.

### Estimation of the actual NPP

The NPP_A_ refers to the actual situation of the grassland NPP, as influenced by climate change and anthropogenic activities and calculated using the CASA model. The NPP is identified by two variables (Zhu, Pan & Zhang, 2007):

(1) \begin{eqnarray*}& NP{P}_{A} \left( x,t \right) =APAR \left( x,t \right) \times \varepsilon \left( x,t \right) \end{eqnarray*}

(2) \begin{eqnarray*}& APAR \left( x,t \right) =SOL(x,t)\times FPAR(x,t)\times 0.5\end{eqnarray*}

where *x* and *t* represent the location and time, respectively. $APAR \left( x,t \right) $ denotes canopy-absorbed incident solar radiation with absorption by pixels *x* at *t* time (MJ m^−2^); ε(*x*, *t*) is the actual light-use efficiency (g C MJ^−1^); and *SOL*(x,t) is the total solar radiation (MJ m^−2^) of pixel *x* at *t* time. *FPAR*(*x*, *t*) represents the fraction of photosynthetically active radiation absorbed by the vegetation canopy of pixel x at time t, and the constant value of 0.5 indicates the fraction of total solar radiation that can be used by vegetation (0.38–0.71 µm). $\varepsilon \left( x,t \right) $ is calculated as follows:

(3) \begin{eqnarray*}\varepsilon \left( x,t \right) ={T}_{\varepsilon 1}(x,t)\times {T}_{\varepsilon 2}(x,\mathrm{t})\times {W}_{\varepsilon }(x,t)\times {\varepsilon }_{\mathrm{max}}\end{eqnarray*}

where *T*_ε1_(*x*, *t*) and *T*_ε2_(*x*, t) are the coefficients of temperature stress; *W*_ε_(*x*, *t*) is the coefficient of water stress; and ε_*max*_ is the maximal light-use efficiency under the most favourable circumstances. Here, the calculation and improvement methods for *T*_ε1_(*x*, *t*), ${T}_{\varepsilon 2} \left( x,\mathrm{t} \right) ,{W}_{\varepsilon }(x,t)$ and ε_*max*_ were provided by Zhu, Pan & Zhang (2007).

### Estimation of the potential NPP

The NPP_P_ reflects the ideal circumstances of the grassland NPP, in which it is only affected by the climate and does not have interference from human activities. It was computed by the Thornthwaite Memorial model, which was derived from the Miami model (Lieth, 1975) and Thornthwaite’s potential evaporation model (Lieth & Box, 1972).

(4) \begin{eqnarray*}& NP{P}_{P}=3000 \left[ 1-{e}^{-0.0009695(V-20)} \right] \end{eqnarray*}

(5) \begin{eqnarray*}& V= \frac{1.05r}{\sqrt{1+{ \left( 1+ \frac{1.05r}{L} \right) }^{2}}} \end{eqnarray*}

(6) \begin{eqnarray*}& L=3000+25t+0.05{t}^{3}\end{eqnarray*}

Here, *NPP*_*P*_ indicates the climate potential NPP (g m^−2^ a^−1^) per year; V represents the real evapotranspiration (mm) per year; L is the maximum evapotranspiration (mm) per year; and *t* and *r* are the average temperature (°C) and precipitation (mm) per year, respectively.

### Estimation of the anthropogenic appropriation of the NPP

The third NPP is the NPP_H_, which is calculated as the NPP_P_ minus the NPP_A_ (Zhou et al., 2017):

(7) \begin{eqnarray*}NP{P}_{H}=NP{P}_{P}-NP{P}_{A}\end{eqnarray*}

**Figure 4:**
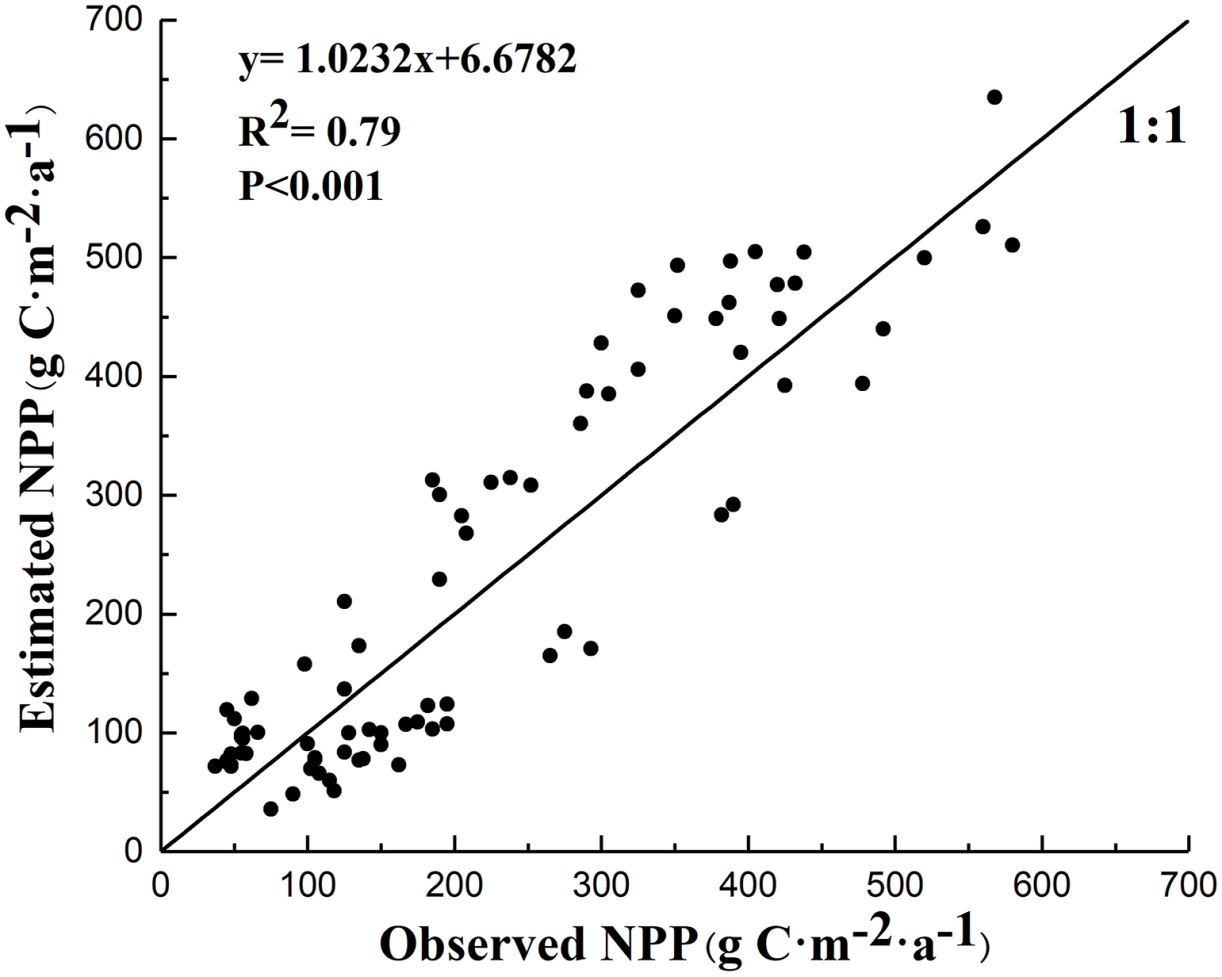
Consistency test between the measured value of the grassland NPP and the simulated value from the CASA model.

The accuracy of *NPP*_*A*_ when calculated by the CASA model was validated by field observation data for grasslands (83 data points) in August 2015. Figure 4 shows the consistency test between the measured value of the grassland *NPP* and the simulated value from the CASA model. The statistical results (*R*^2^ = 0.790, *P* < 0.001) indicated that the CASA model in the study area is reliable.

### Trend analysis of NPP

The Theil-Sen median trend analysis combined with the Mann–Kendall test can be utilized to determine the variation trend of long-term series data (Jiang et al., 2015). We adopted the Theil-Sen median trend analysis method to judge the trends of the grassland NPP_A_, NPP_P_ andNPP_H_ from 2001 to 2015 in the study area. Because regions where the S(NPP) is completely equal to 0 are not observed, this study divided the S(NPP) into five grades according to the actual situation of the S(NPP) (Table 1). We selected the Mann–Kendall method for the significance test. The confidence level was selected as 0.05, and the results were classified according to significant changes (—Z—>= 1.96) and insignificant changes (—Z—<1.96) (Table 1).

**Table 1:** Significance levels of the NPP_A_, NPP_P_ and NPP_H_.

S(NPP_A_), S(NPP_P_)	S(NPP_H_)	$ \left\vert Z \right\vert $	Variation trend of NPP
≥0.0005	≤−0.0005	≥1.96	Significant improvement
≥0.0005	≤−0.0005	≤1.96	Slight improvement
−0.0005 ≤ (S(NPP_A,__P,__H_)≤0.0005)		≤1.96	Stable
≤−0.0005	≥0.0005	≤1.96	Slight degradation
≤−0.0005	≥0.0005	≥1.96	Significant degradation

The trend analysis is calculated by the Theil-Sen median:

(8) \begin{eqnarray*}\mathrm{S}=\text{median} \left( \frac{{x}_{i}-{x}_{j}}{i-j} ,{\forall }_{\mathrm{i}}\lt j \right) \end{eqnarray*}

where 1<*i*<*j*<n and S is the median of the slope of the n (*n* − 1)/2 data combination. When S>0, it reflects that the data shows an upward trend, whereas when S<0, the data shows a declining trend.

The Mann–Kendall test determines whether an increasing or decreasing trend occurs in a time series. The Mann–Kendall test has been diffusely applied in meteorological studies because of its significant advantages of not being affected by a few abnormal values and not requiring samples to follow a certain distribution (Fensholt et al., 2012). We take the *x* value of a given time sequence, and the parameter *Z*_*c*_ as the attenuation index of pixel *x*, and the calculation formula is as follows:

(9) \begin{eqnarray*}& & {Z}_{c}= \left\{ \begin{array}{@{}ll@{}} \displaystyle \frac{S-1}{\sqrt{var \left( S \right) }} &\displaystyle S\gt 0 \\ \displaystyle 0&\displaystyle S=0\\ \displaystyle \frac{S+1}{\sqrt{var \left( S \right) }} &\displaystyle S\text{\lt}0 \end{array} \right. \end{eqnarray*}

(10) \begin{eqnarray*}& & \mathrm{S}=\sum _{i=1}^{\mathrm{n}-1}\sum _{k=n+1}^{n}sign \left( {x}_{k}-{x}_{i} \right) \end{eqnarray*}

\begin{eqnarray*}& & var(s)= \frac{\mathrm{n}(\mathrm{n}-1)(2\mathrm{n}+5)}{18} \end{eqnarray*}

(11) \begin{eqnarray*}& & sign({x}_{k}-{x}_{i})= \left\{ \begin{array}{@{}ll@{}} \displaystyle 1&\displaystyle {x}_{k}-{x}_{i}\gt 0 \\ \displaystyle 0&\displaystyle {x}_{k}-{x}_{i}=0\\ \displaystyle -1&\displaystyle {x}_{k}-{x}_{i}\lt 0 \end{array} \right. \end{eqnarray*}

where *x*_*k*_*andx*_*i*_ denote the time sequence dataset and n is the magnitude of the dataset. *α* represents a significant variation in the time sequence data at the *α* level when $ \left\vert {Z}_{c} \right\vert \gt {U}_{1-\alpha /2}$, where ±*Z*_1−*α*∕2_ is the standard normal deviation. When *α* = 0.01, 0.05, and 0.1, the corresponding Z values are ±2.576, ±1.96, and ±1.645, respectively. In research, the significance level of *α* = 0.05 is generally applied. When $ \left\vert {Z}_{c} \right\vert $>1.96, the confidence level *α*<0.05, and when $ \left\vert {Z}_{c} \right\vert $<1.96, the confidence level *α*>0.05.

### The method of scenario analysis and quantitative assessment

We contrasted the changing trends the three types of NPP to analyse the individual contributions of climate variation and anthropogenic activities to the grassland NPP (Xu et al., 2009). S(NPP_A_), S(NPP_P_) and S(NPP_H_) were employed to quantify the changing trends of the NPP_A_, NPP_P_ and NPP_H_, respectively. The changing trends were calculated by Eq. (8), and six scenarios were established (Table 2). S(NPP_A_)>0 means that the grassland NPP_A_ shows an increasing trend and the grassland is in a recovery state, and vice versa. S(NPP_P_)>0 indicates that climate change promoted the improvement of the grassland NPP, while S(NPP_P_)<0 indicates that climate change led to the decrease of the grassland NPP. S(NPP_H_)>0 means the decline of grassland NPP was caused by human activities, and S(NPP_H_)<0 indicates the increase of the grassland NPP was promoted by human activities.

**Table 2:** Scenarios of the respective influences of climate and anthropogenic activities on NPP_A_ changes in grasslands.

**Scenario**	**S (NPP**_**A**_**)**	**S (NPP**_**P**_**)**	**S (NPP**_**H**_**)**	**Relative roles of climate change (%)**	**Relative roles of anthropogenic activities (%)**	**Description**
Scenario1	>0	>0	>0	100	0	Climate change (100%) contributes the grassland NPP_A_ increase.
Scenario2	>0	<0	<0	0	100	Human activity (100%) contributes the grassland NPP_A_ increase.
Scenario3	>0	>0	<0	$ \frac{ \left\vert S(NP{P}_{P}) \right\vert }{ \left\vert S(NP{P}_{P}) \right\vert + \left\vert S(NP{P}_{H}) \right\vert } $	$ \frac{ \left\vert S(NP{P}_{H}) \right\vert }{ \left\vert S(NP{P}_{P}) \right\vert + \left\vert S(NP{P}_{H}) \right\vert } $	Both climate change and human activity contribute the NPP_A_ increase.
Scenario4	<0	<0	<0	100	0	Climate change (100%) contributes the grassland NPP_A_ decrease.
Scenario5	<0	>0	>0	0	100	Human activity (100%) contributes the grassland NPP_A_ decrease.	
Scenario6	<0	<0	>0	$ \frac{ \left\vert S(NP{P}_{P}) \right\vert }{ \left\vert S(NP{P}_{P}) \right\vert + \left\vert S(NP{P}_{H}) \right\vert } $	$ \frac{ \left\vert S(NP{P}_{H}) \right\vert }{ \left\vert S(NP{P}_{P}) \right\vert + \left\vert S(NP{P}_{H}) \right\vert } $	Both climate change and human activity contribute the NPP_A_ decrease.

Under the condition of S(NPP_A_)>0, three scenarios can be created. Scenario 1: If S(NPP_P_)>0 and S(NPP_H_)>0, then this scenario indicates that the increase in the NPP_A_ is entirely caused by climate change (climate-induced increase, CI). Scenario 2: If S(NPP_P_)<0 and S(NPP_H_)<0, then this scenario means that the increase in NPP_A_ is overall caused by human activities increases (human-induced increase, HI). Scenario 3: If S(NPP_P_)>0 and (S(NPP_H_)<0), this reveals that climate change and anthropogenic activities jointly promoted the enhancement of the grassland NPP_A_ (both induced the increase, BI). When $ \frac{ \left\vert \mathrm{S}({\mathrm{NPP}}_{\mathrm{P}}) \right\vert }{ \left\vert \mathrm{S}({\mathrm{NPP}}_{\mathrm{P}}) \right\vert + \left\vert \mathrm{S}({\mathrm{NPP}}_{\mathrm{H}}) \right\vert } $>$ \frac{ \left\vert \mathrm{S}({\mathrm{NPP}}_{\mathrm{H}}) \right\vert }{ \left\vert \mathrm{S}({\mathrm{NPP}}_{\mathrm{P}}) \right\vert + \left\vert \mathrm{S}({\mathrm{NPP}}_{\mathrm{H}}) \right\vert } $, the increase in the NPP_A_ is mainly caused by climate change, which is expressed as BCI. Conversely, when the increase in the NPP is mainly caused by human activities, this is expressed as BHI. Similarly, under the condition of S(NPP_A_)<0, three scenarios can be created. Under the condition of S(NPP_A_)<0, three scenarios can be created. Scenario 4: If S(NPP_P_)<0 and (S(NPP_H_)<0), then the reduction in the grassland NPP_A_ is caused by climate change (climate-induced decrease, CD). Scenario 5: If S(NPP_P_)>0 and (S(NPP_H_)>0), then the reduction of the NPP in grasslands is entirely due to human activity (human-induced decrease, HD). Scenario 6: If S(NPP_P_)<0 and (S(NPP_H_)>0), then the decrease of the NPP_A_ in grasslands is the result of climate change and anthropogenic activities (both induced the decrease, BD). When $ \frac{ \left\vert \mathrm{S}({\mathrm{NPP}}_{\mathrm{P}}) \right\vert }{ \left\vert \mathrm{S}({\mathrm{NPP}}_{\mathrm{P}}) \right\vert + \left\vert \mathrm{S}({\mathrm{NPP}}_{\mathrm{H}}) \right\vert } $>$ \frac{ \left\vert \mathrm{S}({\mathrm{NPP}}_{\mathrm{H}}) \right\vert }{ \left\vert \mathrm{S}({\mathrm{NPP}}_{\mathrm{P}}) \right\vert + \left\vert \mathrm{S}({\mathrm{NPP}}_{\mathrm{H}}) \right\vert } $, the decrease in the NPP is substantially caused by climate change (BCD), and conversely, the decrease in the NPP is mainly caused by human activities (BHD) when $ \frac{ \left\vert \mathrm{S}({\mathrm{NPP}}_{\mathrm{P}}) \right\vert }{ \left\vert \mathrm{S}({\mathrm{NPP}}_{\mathrm{P}}) \right\vert + \left\vert \mathrm{S}({\mathrm{NPP}}_{\mathrm{H}}) \right\vert } $<$ \frac{ \left\vert \mathrm{S}({\mathrm{NPP}}_{\mathrm{H}}) \right\vert }{ \left\vert \mathrm{S}({\mathrm{NPP}}_{\mathrm{P}}) \right\vert + \left\vert \mathrm{S}({\mathrm{NPP}}_{\mathrm{H}}) \right\vert } $.

### Calculation of the grazing pressure index

To analyse and evaluate the impact of grazing pressure on the NPP_A_ of grassland, this study quantified the grazing pressure by adopting the grazing pressure index (GPI) (Fan, Shao & Liu, 2010). The formula is as follows:

(12) \begin{eqnarray*}GPI= \frac{{C}_{s}}{{C}_{P}} \end{eqnarray*}

where *GPI* is the grazing pressure index and *C*_*s*_ and *C*_*P*_ are the actual and reasonable carrying capacity (sheep units⋅ha^−1^), respectively. If *P*_*i*_ = 1, it implies that the grassland is in balance with livestock. If *P*_*i*_>1, it indicates that the grassland has been overgrazed. If *P*_*i*_<1, then the grassland still has unutilized carrying capacity. The calculation of *C*_*s*_ is as follows:

(13) \begin{eqnarray*}{C}_{s}= \frac{Amount\times {G}_{t}}{Ar\times 365} \end{eqnarray*}

where Amount is the amount of livestock (sheep units) in each township in late June (Fuyun County Bureau of Statistics, 2017) and *G*_*t*_ refers to the utilization time (days) of grassland in different seasons. The grazing periods of summer pastures, spring/autumn pastures, and winter pastures are 90 days, 155 days and 120 days, respectively. *Ar* is the grassland area (ha) of pastures during different seasons in each township. The calculation of *C*_*P*_ is as follows:

(14) \begin{eqnarray*}{C}_{p}= \frac{Y\times U\times C\times H}{S\times D} \end{eqnarray*}

where *Y* is the grass yield per unit area (kg ha^−1^). The grass yield is calculated using the NPP (g C m^−2^a^−1^) obtained by the CASA model, which is then divided by 0.475 and converted into the dry matter mass. Subsequently, the above ground grass yield is calculated according to the ratio coefficient of biomass between the underground biomass and the AGB of different grassland types. In addition, *U* is the grass utilization rate (%), *C* is the available proportion of grassland (%), and *H* is the ratio of edible herbage (%). The values of *U*, *C* and *H* were determined by referring to the standard of the “calculation of reasonable livestock carrying capacity in natural grassland” (NY/t635-2002) and by consulting local experts. *S* is the feed intake per sheep unit per day (kg fresh forage per sheep unit), which is measured as 4 kg fresh forage (Su, Meng & Wu, 2003), the dry weight of fresh herbage is calculated as 1:3, and *D* is the grazing period of the grassland.

### Correlation analysis

In order to analyse the correlation between NPP_A_ and meteorological factors and GPI, the correlation analysis method was selected in this study (Liu et al., 2018a; Liu et al., 2018b).

(15) \begin{eqnarray*}{\mathrm{r}}_{\mathrm{xy}}= \frac{\sum _{\mathrm{i}=1}^{\mathrm{n}} \left[ ({\mathrm{x}}_{\mathrm{i}}-\overline{x})({\mathrm{y}}_{\mathrm{i}}-\overline{y}) \right] }{\sqrt{\sum _{\mathrm{i}=1}^{\mathrm{n}}({\mathrm{x}}_{\mathrm{i}}-\overline{x})^{2}}\sqrt{\sum _{\mathrm{i}=1}^{\mathrm{n}}({\mathrm{y}}_{\mathrm{i}}-\overline{y})^{2}}} \end{eqnarray*}

where r_xy_ is the correlation coefficient between the two variables; x_i_ is the value of the *i*th year of one of the variables, and $\overline{x}$ is the average value of many years; y_i_ is the value of the *i*th year of another variable; $\overline{y}$ is the average value of many years. n is the number of samples. The partial correlation calculation formula is as follows:

(16) \begin{eqnarray*}{\mathrm{r}}_{\mathrm{xy},\mathrm{z}}= \frac{{\mathrm{r}}_{\mathrm{xy}}-{\mathrm{r}}_{\mathrm{xz}}{\mathrm{r}}_{\mathrm{yz}}}{\sqrt{ \left( 1-{\mathrm{r}}_{\mathrm{xz}}^{2} \right) \left( 1-{\mathrm{r}}_{\mathrm{yz}}^{2} \right) }} \end{eqnarray*}

r_xy,z_ is the partial correlation coefficient of *x* and *y* after variable *z* is fixed, that is, the influence of *z* is eliminated in the correlation analysis of *x* and y. Finally, the significance test of the partial correlation coefficient is also needed. The t test method is generally used, and the statistical formula is as follows:

(17) \begin{eqnarray*}\mathrm{t}= \frac{{\mathrm{r}}_{\mathrm{xy},\mathrm{z}}\sqrt{\mathrm{n}-\mathrm{m}-1}}{\sqrt{1-{\mathrm{r}}_{\mathrm{xy},\mathrm{z}}^{2}}} \end{eqnarray*}

where r_xy,z_ is partial correlation coefficient; *n* is the number of samples; *m* is the number of degrees of freedom.

## Results

### Interannual variation trends of the grassland NPP

**Figure 5:**
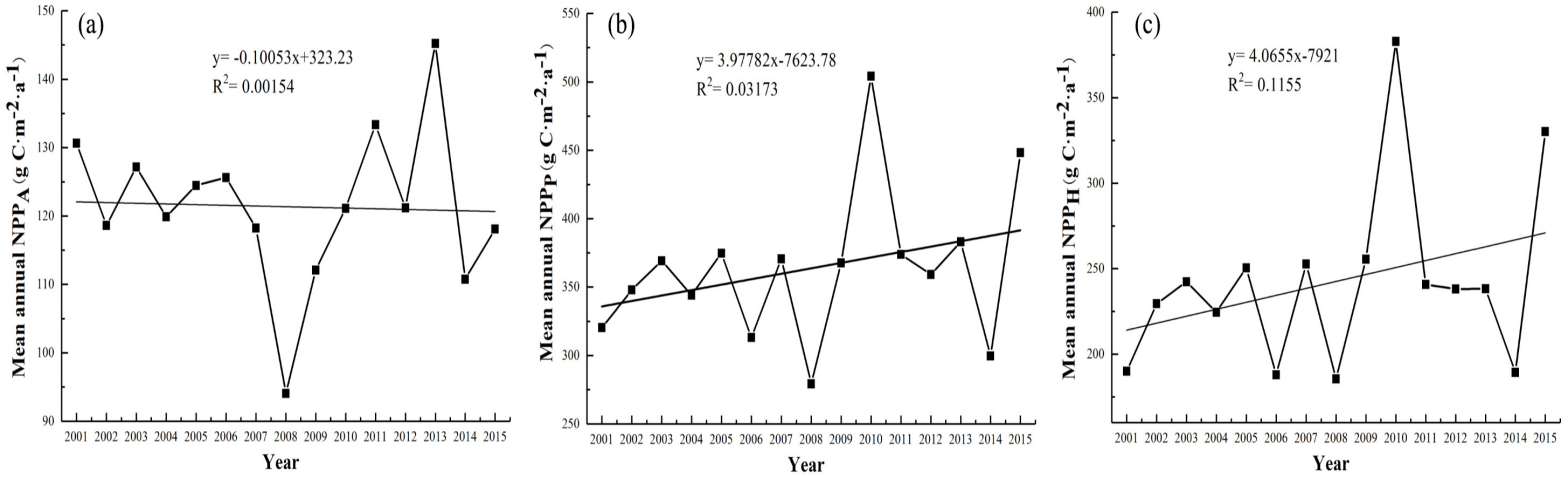
Interannual variation trends of the grassland NPP from 2001–2015. (A) NPP_A_, (B) NPP_P_, and (C) NPP_H_.

The average values of NPP_A_, NPP_P_ and NPP_H_ in grasslands fluctuated greatly between the years 2001 and 2015 (Fig. 5). The lowest values of the regional average NPP_A_, NPP_P_ and NPP_H_ all appeared in 2008 (94.05, 279.24 and 185.49 g C m^−2^ a^−1^, respectively). The maximum value of the NPP_A_ appeared in 2013 (145.23 g C m^−2^ a^−1^), and the maximum values of the NPP_P_ and NPP_H_ both appeared in 2010 (504.18 and 382.85 g C m^−2^ a^−1^, respectively). In general, the average value NPP_A_ showed a slight decreasing trend from 2001 to 2015. From 2001–2008, the grassland NPP_A_ showed a fluctuating downward trend, and the average NPP_A_ value of grasslands decreased from 130.65 g C m^−2^ a^−1^ to 94.05 g C m^−2^ a^−1^. The average NPP_A_ value of grasslands showed a fluctuating increasing trend in 2008–2013. The grassland NPP_P_ showed a fluctuating increasing trend in 2001–2015, and the average NPP_P_ value of grasslands increased from 320.28 g C m^−2^ a^−1^ to 448.26 g C m^−2^ a^−1^ from 2001–2015 (Fig. 5B). Figures 5B and 5C show that the annual variation trend of the NPP_H_ was similar to that of the NPP_P_.

### Spatial differentiation characteristics of the NPP variation trend

**Figure 6:**
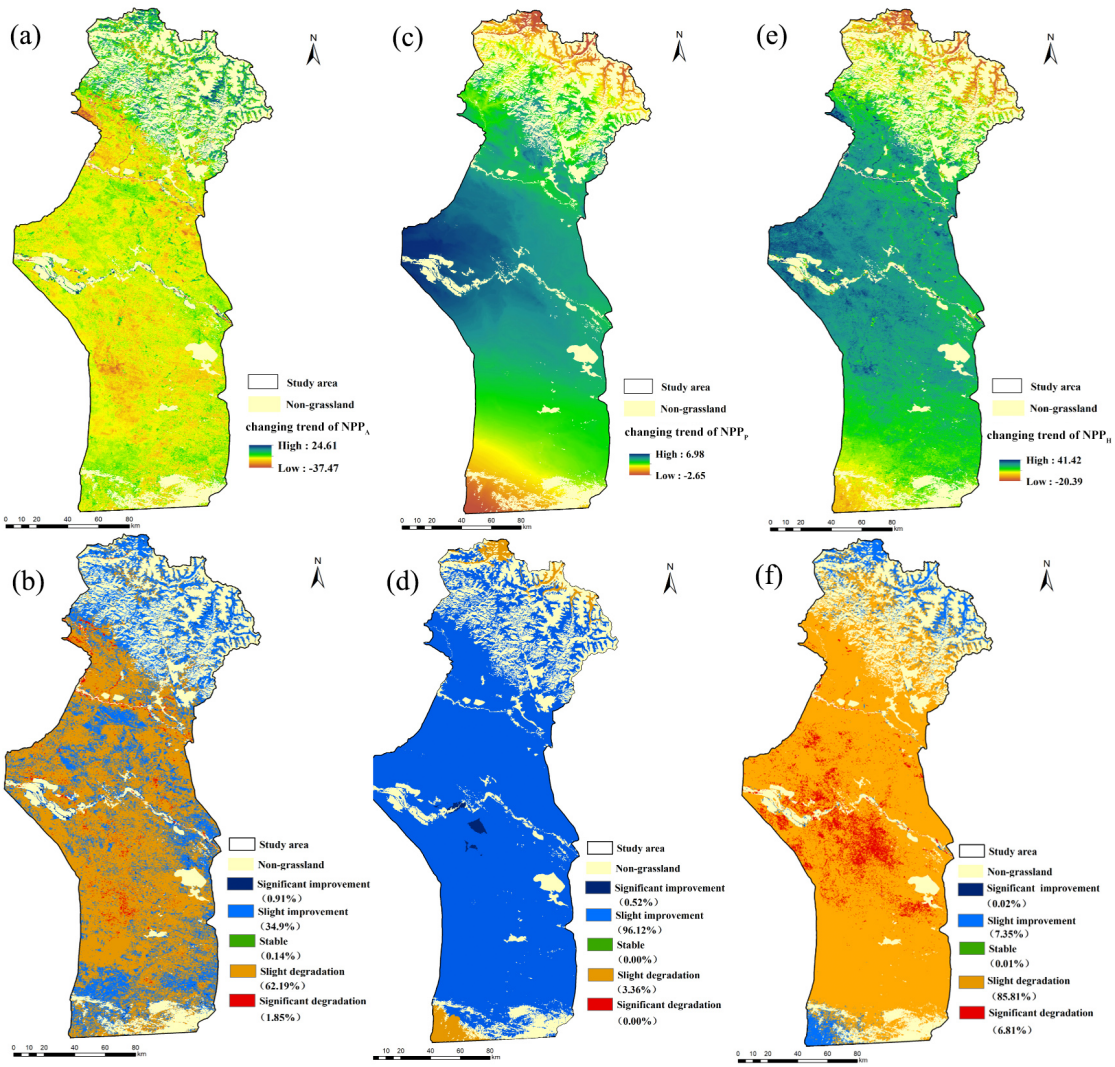
Spatial distributions of grassland NPP variation trend at various significance levels from 2001–2015. (A), (C) and (E) are the changing trends of the NPP_A_, NPP_P_ and NPP_H_; (B), (D) and (F) correspond to their significance levels, respectively.

The changing trend of the NPP_A_ in Fuyun County from 2001 to 2015 is shown in Fig. 6A. The results revealed that the *S*(NPP_A_) of the actual NPP_A_ from 2001 to 2015 was between −37.47 and 24.61 g C m^−2^ a^−1^. The improvement area of the grassland NPP_A_ occupied 35.81% of the total grassland area, and the improvement lay mostly in the northern mountainous region of the investigated area. Areas with no significant changes occupied 0.14% of the total grassland area. The area of degraded grassland NPP_A_ occupied 64.05% and was predominately located in the central and southern zones of the investigation area (Fig. 6B).

The *S*(NPP_P_) in the study area from 2001 to 2015 was between −2.65 and 6.98 g C m^−2^ a^−1^. As shown in Fig. 6C, the NPP_P_ of grassland was estimated by the climate productivity model and was generally increasing. The NPP_P_ of grassland displayed a rising trend in nearly 96.64% of the study area. Only in 3.36% of the area did the grassland NPP_P_ show a slight downward trend, which occurred in the southwestern corner of the investigation area and the northernmost alpine meadow area (Fig. 6D).

The *S*(NPP_H_) caused by human activities was between −20.39 and 41.42 g C m^−2^ a^−1^ (Fig. 6E). Judging by the *S*(NPP_H_) (Fig. 6E), only 7.37% of the grassland NPP_H_ experienced an increasing trend. This increase lay mostly in the northernmost high-altitude mountains of the study area and belonged to summer pasture, indicating that human activities produced positive influences on the growth of grassland in these areas. Furthermore, almost 92.62% of grassland displayed a downward tendency of the grassland NPP_H_, and this decrease was mainly located in spring/autumn pastures and winter pastures, which meant that human activities were detrimental to grassland growth in these areas (Fig. 6F).

### Influence of climate change and anthropogenic activities

**Figure 7:**
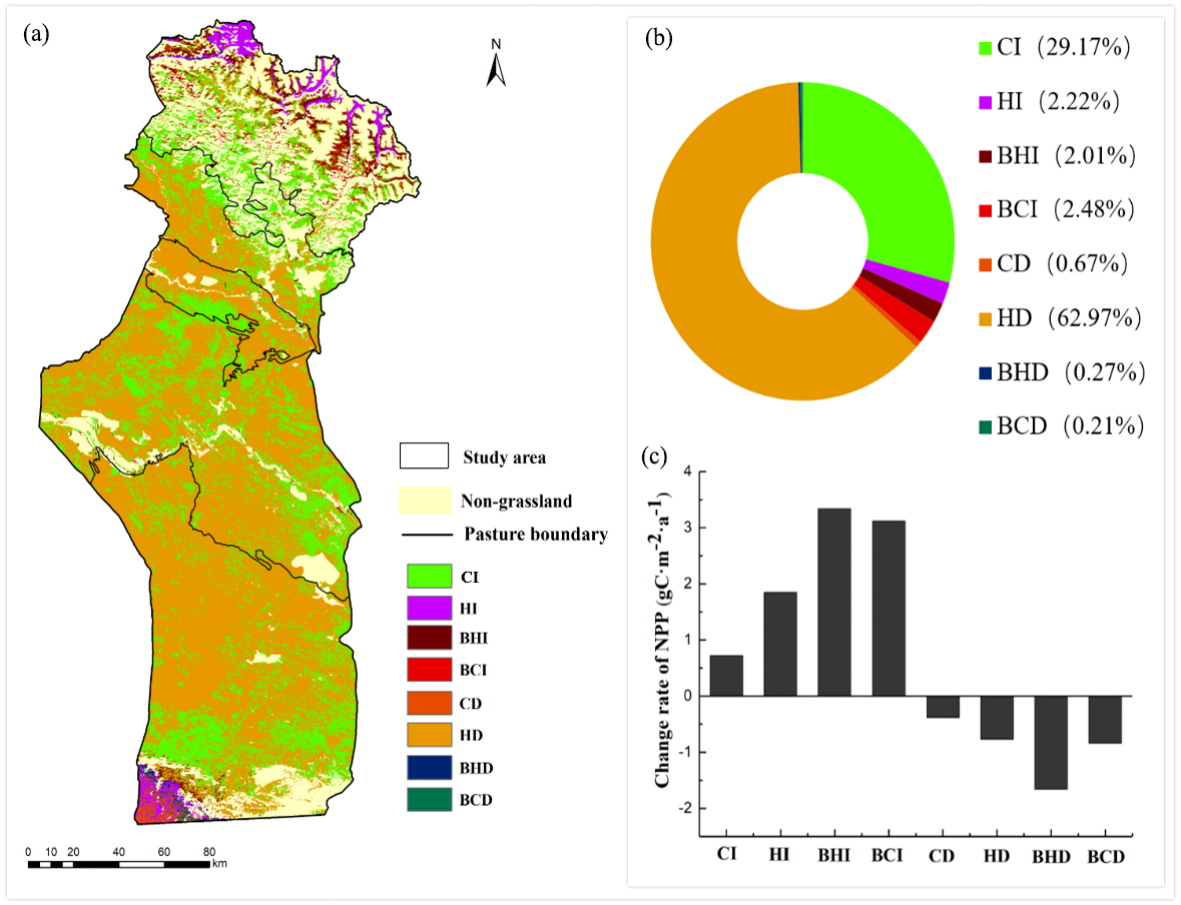
Spatial distribution of the individual impacts of climate change and anthropogenic activities on grasslands from 2001–2015 (A); Percentage of the relative effect (B) and change rates of the NPP caused by different factors (C). CI, the NPP_A_ increase was entirely caused by climate change; HI, the NPP_A_ increase was entirely caused by anthropogenic activities increasing; BHI, the NPP_A_ increase was mainly caused by anthropogenic activities; BCI, the NPP_A_ increase was mainly caused by climate change; CD, the NPP_A_ decrease was entirely induced by climate change; HD, the NPP_A_ decrease was entirely induced by anthropogenic activities; BHD, the NPP_A_ decrease was mainly caused by anthropogenic activities; BCD, the NPP_A_ decrease was mainly induced by climate change.

The relative roles of climate change and human activities in grassland NPP degradation or restoration were assessed based on the methods listed in Table 2. As shown in Fig. 7A, the spatial distribution of changes in the grassland NPP_A_ caused by climate change, anthropogenic activities, and their combined effects from 2001–2015 were identified. Overall, the results indicated that the grassland NPP_A_ area showing an increasing trend (35.88%) was smaller than that exhibiting a decreasing trend (64.12%).

**Figure 8:**
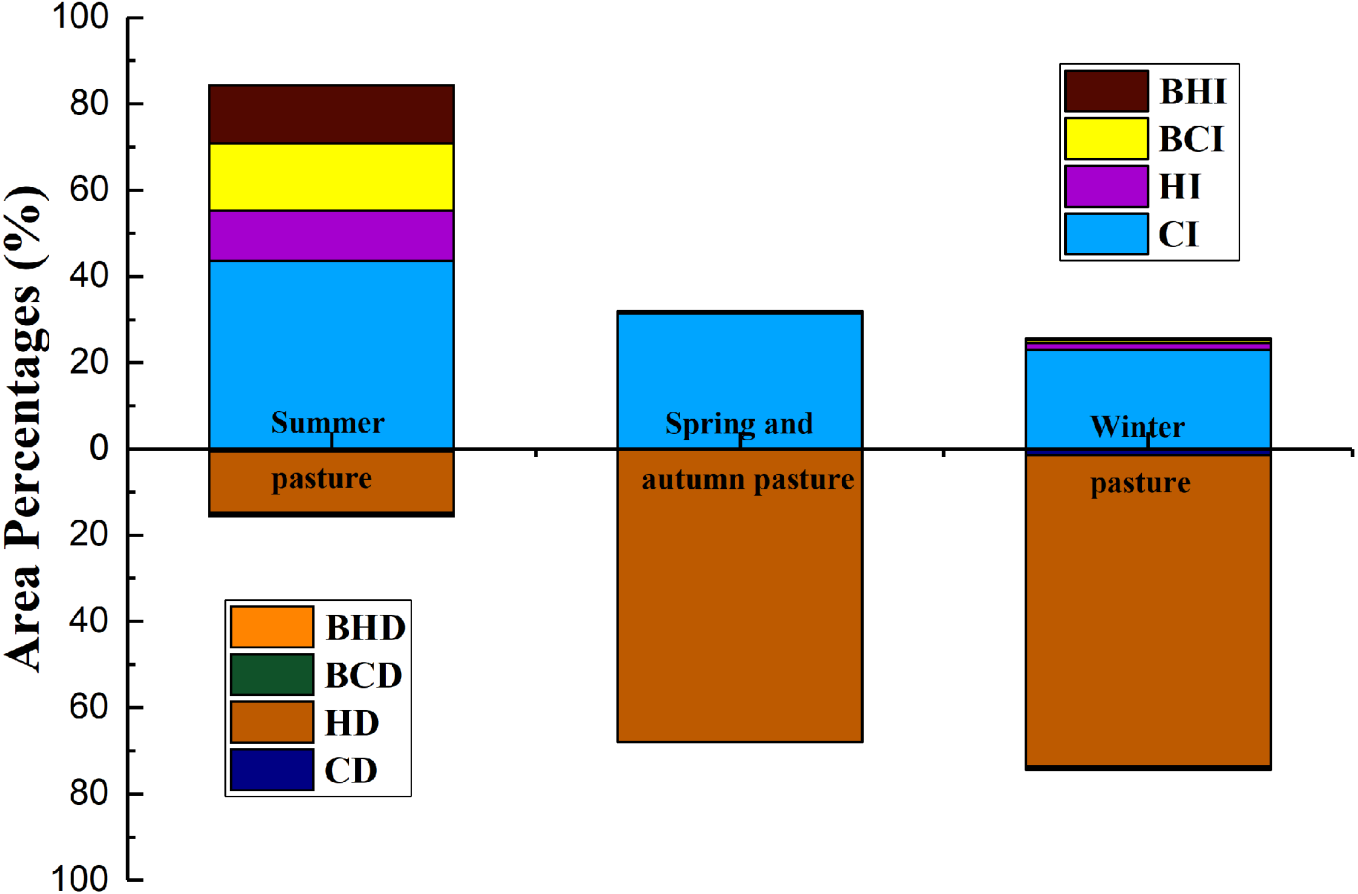
NPP_A_ change caused by climate change and anthropogenic activities in different seasonal grazing pastures from 2001–2015.

From 2001 to 2015, the area of grassland NPP_A_ with an increasing trend was 9,562.41 km^2^, occupying 35.88% of the grassland area in the research area. The area where the grassland NPP_A_ has increased entirely as a result of climate change (CI) was 7,775.61 km^2^, occupying 81.30% of the area of increased NPP_A_, which was concentrated in the southern part of the summer pasture. The area of NPP_A_ increase that occurred entirely due to anthropogenic activities (HI) was 591.05 km^2^, occupying only 6.19% of the area of increased NPP_A_, which was mostly found in the northernmost zones of the investigation area. Overall, the regions where anthropogenic activities and climate change together (BCI+BHI) led to an improvement of the grassland NPP were concentrated in the middles of summer pastures, which occupied 12.51% of the entire area with an NPP_A_ increase (Figs. 7A, 7B and 8). Among them, the rate of increase of the NPP_A_ in the BHI region was the fastest, with an increasing tendency rate of 33.4 gC m^−2^ 10a^−1^ (Fig. 7C).

The area of grassland NPP_A_ with a decreasing trend was 17,093.92 km^2^, occupying 64.12% of the grassland area in the research area. The total area with a NPP_A_ decrease caused entirely by climate change (CD) was only 178.33 km^2^, accounting for 1.04% of the area of decreased NPP_A_. The total area of NPP_A_ decrease entirely caused by human activities (HD) was 16,788.57 km^2^, which was mostly located in the spring/autumn pastures and summer pastures in the central and southern zones of the study area, accounting for 98.21% of the area of decreased NPP_A_ (Figs. 7A, 7B and 8). The joint influence of climate change and anthropogenic activities have only caused a 0.75% decline in the grassland NPP_A_ (BCD + BHD) (Figs. 7A and 7B). Among them, the NPP_A_ decreased fastest in the BHD region, where it presented a decreasing tendency rate of 16.6 gC m^−2^ 10a^−1^ (Fig. 7C).

### Comparative analysis of the driving contributions of two factors in the different seasonal pastures

For summer pastures, the area in which the grassland NPP_A_ showed an increasing trend was much larger than the area of decrease. In total, 84.33% of grassland area showed an increase in the NPP_A_, while nearly 15.67% of the grassland area showed a decrease in the NPP_A_. The areas where the grassland NPP_A_ has increased due to the BHI+BCI, HI and CI occupied 34.50%, 13.87% and 51.63% of the total increased NPP_A_ area, respectively. For NPP_A_ decrease, the decreased grassland NPP_A_ area caused by HD occupied 90.75% of the total decreased NPP_A_ area (Fig. 8). For spring/autumn and winter pasture, 32.02% and 25.60% of the grassland area showed NPP_A_ increases, while 67.98% and 74.40% of the grassland area presented NPP_A_ decreases in the spring/autumn and winter pasture, respectively. Most of the increased NPP_A_ areas were due to CI, and most of the decreased NPP_A_ areas were due to HD (Fig. 8).

### Variation in the grassland pressure index and the correlation analysis with the grassland NPP

**Figure 9:**
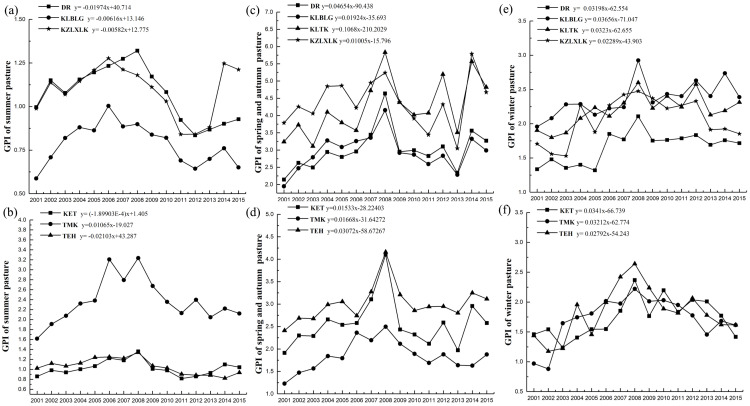
Interannual variation of the grazing pressure index in the different seasonal pastures of different townships. (A) and (B) are the summer pastures; (C) and (D) are the spring and autumn pastures; (E) and (F) are the winter pastures.

**Figure 10:**
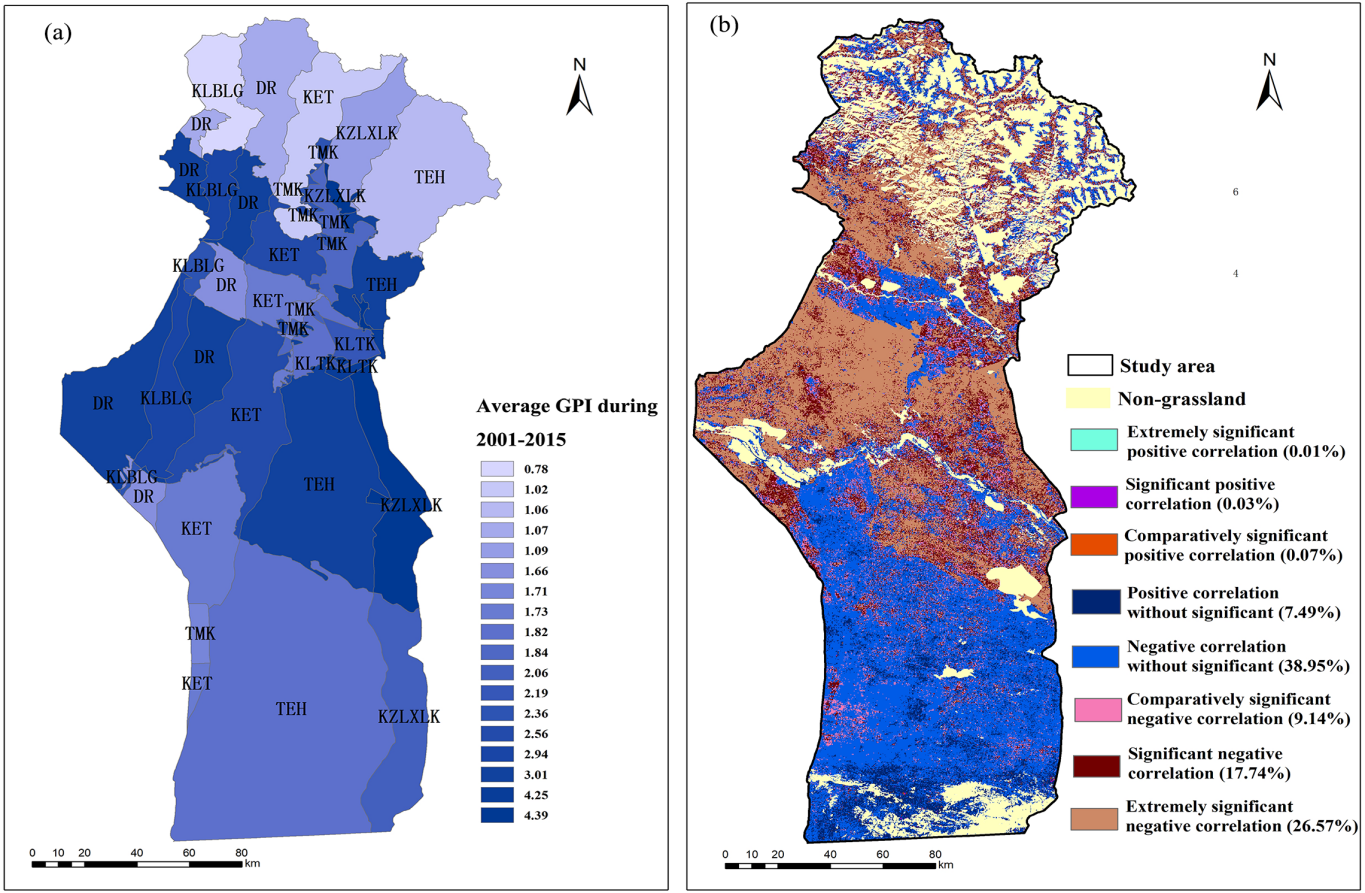
Spatial distributions of the average GPI from 2001–2015 (A); significance test of the correlation coefficients between the annual grassland NPP_A_ and the annual average GPI (B). Extremely significant positive correlation *P* < 0.01; significant positive correlation *P* < 0.05; comparatively significant positive correlation *P* < 0.10; positive correlation without significant *P* > 0.10; extremely significant negative correlation *P* < 0.01; significant negative correlation *P* < 0.05; comparatively significant negative correlation *P* < 0.10; negative correlation without significant *P* > 0.10.

The information provided by the Fuyun County grassland station showed that most of the townships in Fuyun County are distributed in a long north-south strip, meaning that each township area contains different seasonal pastures (Fig. 3B). To calculate the pressure of carrying livestock on the grassland, the livestock in each township was considered to graze in their respective administrative areas. Figures 9A and 9B show the temporal dynamic change of GPI in summer pasture in each township (there is no summer pasture in KLTK town) in the study area from 2001 to 2015. The average GPI values of the summer pastures of DR, KLBLG, KZLXLK, KET, TMK and TEH were 1.07, 0.78, 1.09, 1.02, 2.36 and 1.06, respectively (Fig. 10A). The GPI change rates of summer pastures in DR, KLBLG, KZLXLK and TEH towns were negative (−0.197 ⋅10a^−1^, −0.062 ⋅10a^−1^, −0.0582 ⋅10a^−1^ and −0.2103 ⋅10a^−1^, respectively). In contrast, the GPI change rates of summer pastures in KET and TMK towns were close to 0 and 0.1065 ⋅10a^−1^, respectively.

The average GPI values of the spring and autumn pasture of DR, KLBLG, KLTK, KZLXLK, KET, TMK and TEH were 3.01, 2.94, 4.25, 4.39, 2.56, 1.84 and 3.01, respectively (Figs. 9C, 9D and 10A). The GPI values of winter pastures were 1.66, 2.36, 2.19, 2.06, 1.73, 1.71 and 1.82, respectively (Figs. 9E, 9F and 10A). In addition, the slope change of the GPI in all towns for spring/autumn pastures and winter pastures was positive over the past 15 years.

According to the weight of the area and the GPI of summer pastures, spring/autumn pastures and winter pastures in each township, the 15-year average GPI was calculated for summer pastures, spring/autumn pastures and winter pastures. The average GPI values of summer pastures, spring/autumn pastures and winter pastures were 1.04, 3.03 and 1.83, respectively. In addition, the correlation between the NPP_A_ and GPI was explored by Eqs. (15) and (17) (Fig. 10B). The positive-correlation area accounted for 7.59% of the total area, and the negative-correlation area accounted for 92.41% of the total area. Among the spring and autumn pastures, 98.97% were negatively correlated with the GPI. Among the winter pastures and summer pastures, 85.12% and 96.02% were negatively correlated with the GPI, respectively. The areas with negative correlations, comparatively significant negative correlations, significant negative correlations and extremely significant negative correlations accounted for 13.22%, 8.53%, 24.52% and 52.70% of the spring and autumn pasture area; 65.72%, 8.54%, 8.49% and 2.36% of the winter pasture area, and 30.98%, 13.27%, 27.48% and 24.29% of the summer pasture area, respectively.

## Discussion

### Methodology

The traditional grassland dynamic monitoring method relies heavily on field surveys. Field investigations cost an enormous amount of manpower and resources and are inefficient, particularly, large-scale grassland surveys (Zhou et al., 2017). It is much more effective to assess grassland degradation by remote sensing (Lu et al., 2007).

We chose the NPP as an evaluation index of grassland dynamic variation (Fang et al., 2013). The CASA model and Thornthwaite Memorial model were selected to simulate the grassland NPP_A_ and NPP_P_, respectively. It should be noted that the CASA model and Thornthwaite Memorial model might include estimation error which would bring uncertainty to the estimated NPP_A_ and NPP_P_ results. In addition, climate change and anthropogenic activities were believed to be the major factors affecting the changes of grasslands overall (Mu et al., 2013). Many researchers have studied the relative influence of anthropogenic activities and climate variation on grassland degradation by comparing the grassland NPP_A_ with the grassland NPP_P_ (Jiao et al., 2018; Zheng et al., 2019), and the NPP_H_ has been used to evaluate the impact of anthropogenic activities on the grassland ecosystem (Xu et al., 2010).

This approach was based on the hypothesis that grassland dynamic variations were determined only by climate change and anthropogenic activities, and the driving factors of grassland dynamic changes can be quantitatively evaluated by scenario simulations (Yang et al., 2016). Although the NPP_P_ and NPP_H_ have successfully been applied in the assessment of grassland dynamic changes (Zhou et al., 2015; Zheng et al., 2019), this methodology has its drawbacks (Yang et al., 2016). First, the NPP_P_ represents that the changes in the grassland NPP are affected merely by temperature and precipitation under ideal conditions. Second, we evaluated the respective influences of climate change and anthropogenic activity on NPP changes and established a scenario assuming that climate change and anthropogenic activities were the only driving factors of NPP variation in grasslands. Nevertheless, this situation is additionally influenced by other factors (such as grassland rodents, grassland species and grassland fires), which have not been quantified in this research due to the limits of our research methods. These factors would likely introduce uncertainty into the research results. In future studies, other factors should be considered when assessing dynamic changes in the grassland NPP. However, the current analysis results quantitatively distinguished the relative influences of climate change and anthropogenic activities on grassland NPP changes and indicated the effects of grazing activities on the variation in the NPP in grasslands, which provides a solid foundation for further analyses.

### Climate change and its influence on the grassland NPP

Grassland ecosystems are vulnerable to climate change, especially in arid regions (Lioubimtseva, 2004). Climate change could directly influence grassland growth through precipitation and temperature, which can affect the soil moisture and microbes, and determine the grassland growth and ecosystem productivity under different hydrothermal conditions (He et al., 2015). The results showed that 81.30% of the increase in the grassland NPP_A_ was due to climate change (CI), which indicated that climatic circumstances played a significant role in NPP_A_ changes. To identify which climatic factor has the greatest impact on grassland changes, a partial correlation was performed to analyse the response of NPP_A_ to temperature and precipitation changes, and the significance test of partial correlation coefficient was carried out by t test.

**Figure 11:**
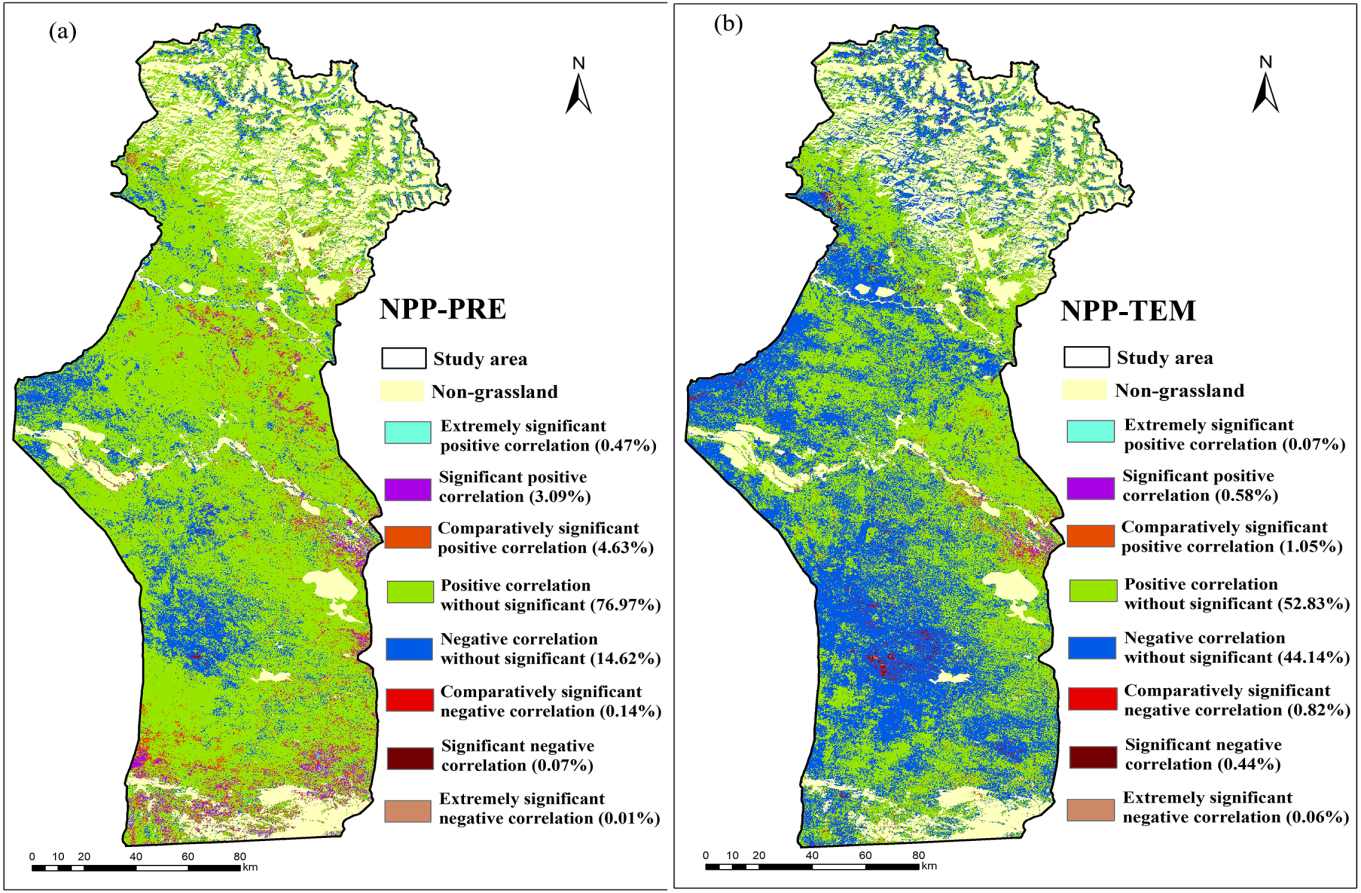
Spatial distributions of the significance test of partial correlation coefficients between the annual NPP_A_ and climatic factors. (A) Significance test of partial correlation coefficients between the annual grassland NPP_A_ and annual precipitation; and (B) significance test of partial correlation coefficients between the annual grassland NPP_A_ and annual average temperature. Extremely significant positive correlation P1/20.01; significant positive correlation *P* < 0.05; comparatively significant positive correlation *P* < 0.10; positive correlation without significant *P* > 0.10; extremely significant negative correlation *P* < 0.01; significant negative correlation *P* < 0.05; comparatively significant negative correlation *P* < 0.10; negative correlation without significant *P* > 0.10.

The area in which the grassland NPP_A_ was positively correlated with precipitation accounted for 85.16% of the total area (Fig. 11A). This indicated that the grassland NPP_A_ was susceptible to precipitation changes, because precipitation reduction depleted the soil moisture, causing drought that reduced the photosynthetic efficiency (Fensholt et al., 2012). Therefore, the increase of precipitation was the leading factor behind the increase of the grassland NPP_A_ in the study area. In addition, the precipitation trend rate analysis showed that the average annual precipitation in the study area presented an increasing trend (Fig. 2A), so the potential NPP_P_ of most areas of the grassland (96.64%) presented an increasing trend (Fig. 6D). This finding is in agreement with that of Liang et al. (2015), who found that precipitation mostly controlled the vegetation NPP in arid and semi-arid regions. Zhang & Ren (2017) reported that atmospheric precipitation was the main source of moisture for vegetation in the arid ecosystem in Central Asia. Precipitation generally increases the soil moisture content so that the soil can provide more water to vegetation and enhance the photosynthesis of vegetation, thus increasing the NPP of vegetation (Zheng et al., 2006). In contrast, negative correlations between the grassland NPP_A_ and precipitation accounted for 14.84% of the study area, and negative responses of the grassland NPP_A_ to precipitation alteration were found in high-altitude mountains (Fig. 11A). The photosynthesis of vegetation would be inhibited due to the decrease of radiation and the increase of the relative humidity when the increase of precipitation exceeds the needs of vegetation growth (Ukkola et al., 2016). In addition, excessive precipitation will reduce the soil organic matter, which will aggravate soil erosion and flooding, and destroy plant habitats (Qu et al., 2018).

The correlation between the NPP_A_ and temperature was also studied (Fig. 11B). Positive correlations occupied 54.53% of the study area, while negative correlations occupied 45.46% of the study area (Fig. 11B). The response of the NPP_A_ in grassland to temperature change did not follow an obvious regional distribution rule. In addition, there were nearly no significant correlations between the grassland NPP_A_ and temperature. This finding was also reported by Chen et al. (2019), who noted that the grassland NPP was insensitive to temperature change and that temperature may only play a nonessential role in the growth of grassland in most parts of Central Asia. Additionally, Xu, Wang & Yang (2017) found that grassland and desert vegetation responded more strongly to precipitation than to temperature in summer.

Note that there are few regions with significant correlation between NPP_A_ and temperature and precipitation by the statistical analysis (Fig. 11), which was consistent with Zhou, Li and Zhu (2015)’s findings in Xinjiang. This may be related to the fact that we conducted the partial correlation analysis of NPP with temperature and precipitation on an annual scale. Previous studies have shown that the significant correlation between vegetation and temperature and precipitation appeared in the statistics of seasonal scale or monthly scale (Zhang et al., 2011; Xu, Wang & Yang, 2017).

In addition, the inter-annual variation of the NPP_A_ and NPP_P_ from 2001–2015 was consistent with precipitation but inconsistent with temperature. The lowest values of the regional average NPP_A_ and NPP_P_ both occurred in 2008 (Fig. 5), which were probably due to the lowest precipitation occurring in 2007 and 2008 (Fig. 2). This finding suggests that grassland growth responded more strongly to precipitation than to temperature (Yang et al., 2016). Hence, precipitation is a decisive aspect that affects grassland growth, particularly in arid regions (Herrmann, Anyamba & Tucker, 2005).

### Impact of human activities on the grassland NPP

Several reports have shown that anthropogenic activities were the major driving forces of grassland dynamic variations (Yang et al., 2016; Chen et al., 2017). We recognised that human activities contributed 65.19% of the grassland NPP_A_ changes in the investigate area, and the human-induced increase and human-induced decrease in the grassland NPP_A_ occupied 2.22% and 62.97% of the whole region, respectively. In addition, the research findings showed that 100% and 97% of the grassland degradation was due to anthropogenic activities in spring/autumn pastures and winter pastures, respectively. This result was consistent with Chen et al. (2019), who noted that desert grasslands were more vulnerable to human activities than were alpine grasslands in Central Asia.

Grazing was regarded as the main driving force of grassland change induced by anthropogenic activities (Chen et al., 2014). In summer pasture, we found that the area of grassland NPP_A_ increased by human activities (HI+BHI) was larger than the area decreased by human activities (HD+BHD) in the towns in which the change rate of the GPI was negative (DR, KLBLG, KZLXLK and TEH). On the contrary, the area of grassland NPP_A_ that was reduced by human activities above the area of grassland NPP_A_ enhanced by human activities occurred in towns where the change rate of the GPI was positive (TMK) (Table S2; Figs. 9A and 9B). Areas of increased grassland NPP_A_ caused entirely by human activities (HI) were located in the northernmost high-altitude mountains of the study area (Fig. 7A), and this was also the area in Fig. 6F where NPP_H_ improved slightly. This area belonged to summer pasture with high altitudes which were difficult to reach for humans and livestock, and few grazing activities were observed at these sites; thus, these grasslands were rarely disturbed by human activities and gradually recovered. The reduced grazing activities may be one of the reasons behind the increase in the grassland NPP, which may have also been related to the implementation of grassland ecological protection engineering measures in Xinjiang (Wang et al., 2014). Areas of grassland where human activities and climate change (BI+CI) have combined to cause an increase in the NPP_A_ were concentrated in the middle of summer pastures. The areas where the NPP_A_ increased in summer pastures entirely due to climate change (CI) were concentrated in the south of summer pastures (Fig. 7A). This area belongs to the low-altitude area of summer pastures, where grazing activities are frequent. Grazing activities have led to the decline in the grassland NPP; hence, the increase in the NPP_A_ in this area was completely caused by climate change.

The areas with slight and significant degradation of NPP_H_ accounted for 85.81% and 6.81% of the study area, respectively, which were distributed in the spring/autumn pasture and winter pasture in the study area (Fig. 6F). For spring and autumn pastures, the area of decreased NPP_A_ in spring and autumn pastures of all towns was larger than the area of increased NPP_A_, except in TMK town, and the degradation of spring and autumn pastures was entirely caused by human activities (Table S3). The utilization time of spring and autumn pastures was up to 155 days, and the utilization period was the key period for spring pasture germination and autumn forage seeding. However, the excessive application of human activities affected the growth and reproduction of pasture areas. Similarly, the proportion of the area of decreased NPP_A_ in winter pastures in all towns was much larger than that of increased grassland NPP_A_ (Table S4). The results showed that the degradation of spring/autumn pastures and winter pastures was almost entirely caused by human activities. The average overloaded grazing rate of spring/autumn pastures and winter pastures in the past 15 years reached 203% and 83.5%, respectively. The slopes of the GPI changes in winter pasture and spring/autumn pasture of all towns were positive, indicating that the GPI has increased in the past 15 years.

In addition, we explored the correlation between average NPP_A_ and average GPI from 2001 to 2015. The negative correlations between the NPP_A_ and GPI occupied most of the research area (92.41%) (Fig. 10B), which indicated that the increase in GPI was one of the most important drivers causing the decrease in the NPP_A_ in grasslands caused by anthropogenic activities. In the spring and autumn pastures, there was an extremely significant negative correlation between the NPP_A_ and GPI, which accounted for 52.71% of the pasture area, indicating that the increase of the GPI led to the degeneration of the grassland NPP, which was particularly prominent in the spring and autumn pastures.

## Conclusion

This study selected the NPP_A_, NPP_P_ and NPP_H_ as indexes to measure grassland dynamic variations in a mountain basin system of Xinjiang, and quantitatively analysed the respective influences of climate change and anthropogenic activities on grassland variations. The conclusions of this study can be summarized as follows:

(1) Experimental results indicated that the NPP_A_ displayed a slight downward trend from 2001 to 2015. In the study area, 35.88% of the grassland experienced an increase in the NPP_A_ while 64.12% of the grassland showed a decrease in the NPP_A_ from 2001 to 2015.
(2) For the areas where the grassland NPP has increased, the respective contributions of climate change and anthropogenic activities were 81.30% and 6.19%. Precipitation was the dominant climatic factor than temperature that controlling the grassland NPP changes in the study area. Anthropogenic activities were the primary cause for the reduction in the grassland NPP_A_, with the region undergoing human-induced decreases occupying 98.21% of the total area undergoing decreases. Human-dominated NPP_A_ decreases were found to occur mainly in spring/autumn pasture and winter pasture.
(3) The average GPI values of summer pasture, spring/autumn pasture and winter pasture were 1.04, 3.03 and 1.83, respectively. Summer pastures were basically in a state of balance between grass and livestock. The spring/autumn pastures were in an extremely unbalanced state between grass and livestock due to a small carrying capacity. Additionally, negative correlation between NPP and GPI occupied most of the whole research area, especially the spring and autumn pasture.

Therefore, the driving force of degradation was human activities, and we should take measures to reduce the intensity of grazing. The implementation of the herdsman settlement project, the provision of vocational training programs to increase the diversify of herdsmen’s livelihoods, the ability to rear farm animals in hut and the construction of related infrastructure are options that can be used to reduce the livestock pressure. In addition, implementing high-quality and high-yield artificial forage grass along the Irtysh River near the spring/autumn pastures and Ulungur River near the winter pastures which can provide stable and sufficient forage for livestock is recommended to reduce the grazing pressure of spring/autumn pastures and winter pastures. Our findings could be useful for pasture management and grassland degradation prevention in MBSs in arid regions.

##  Supplemental Information

10.7717/peerj.9797/supp-1 Data S1 Climate data and NPP data

10.7717/peerj.9797/supp-2 Supplemental Information 2 Supplemental Tables

## Additional Information and Declarations

### Data Availability

The following information was supplied regarding data availability: The raw measurements are available in the Supplemental Files.
