# Supplementary material for: Response of grassland productivity to climate change and anthropogenic activities in arid regions of Central Asia
Source: PeerJ. 2020 Aug 31;8:e9797. doi: 10.7717/peerj.9797 (PMC7469937; doi:10.7717/peerj.9797)
Supplement: Supplemental Information 2 [file peerj-08-9797-s002.doc]

**Supplementary Material**

**Response of grassland dynamicsproductivity to climate change and anthropogenic activities in arid regions of Central Asia: a case study in Fuyun County, China**

*Xu Bi, Bo Li, Lixin Zhang, Zihan Yang, Xinshi Zhang*

**Table 1 Statistics table of measured aboveground biomass**

| Site | Longitude | Latitude | Elevation | Dominant species | Grassland types | Aboveground biomass (g/m^2^) |
| --- | --- | --- | --- | --- | --- | --- |
| 1 | 89.40841 | 46.850738 | 999 | *Seriphidium terrae-aibae,Stipa glareosa* | Temperate desert | 33.83 |
| 2 | 88.75903 | 46.769587 | 726 | *Artemisia desertorum,Seriphidium terrae-aibae* | Temperate desert | 37.59 |
| 3 | 88.64111 | 46.678317 | 726 | *Anabasis brevifolia* | Temperate desert | 86.47 |
| 4 | 88.55078 | 46.698626 | 709 | *Anabasis aphylla* | Temperate desert | 36.09 |
| 5 | 88.47616 | 46.689804 | 655 | *Krascheninnikovia latens,Anabasis brevifolia* | Temperate desert | 31.02 |
| 6 | 88.37451 | 46.703562 | 601 | *Haloxylon ammodendron* | Temperate desert | 59.83 |
| 7 | 88.36884 | 46.699489 | 603 | *Halocnemum strobilaceum* | Temperate desert | 67.67 |
| 8 | 88.27845 | 46.543175 | 652 | *Anabasis brevifolia,Stipa glareosa* | Temperate desert | 102.49 |
| 9 | 88.41794 | 46.558831 | 652 | *Seriphidium terrae-aibae,Stipa glareosa* | Temperate desert | 147.01 |
| 10 | 88.53395 | 46.560326 | 709 | *Seriphidium terrae-aibae,Stipa glareosa* | Temperate desert | 141.56 |
| 11 | 88.42505 | 46.460819 | 717 | *Krascheninnikovia latens,Anabasis brevifolia* | Temperate desert | 102.49 |
| 12 | 88.51527 | 46.472446 | 703 | *Seriphidium terrae-aibae,Stipa glareosa* | Temperate desert | 110.71 |
| 13 | 88.73419 | 46.458329 | 722 | *Anabasis aphylla* | Temperate desert | 74.79 |
| 14 | 88.79977 | 46.44915 | 726 | *Seriphidium terrae-aibae,Stipa glareosa* | Temperate desert | 56.51 |
| 15 | 88.96461 | 46.515204 | 799 | *Seriphidium terrae-aibae,Stipa glareosa* | Temperate desert | 30.47 |
| 16 | 88.90818 | 46.398811 | 779 | *Seriphidium terrae-aibae,Stipa glareosa* | Temperate desert | 58.17 |
| 17 | 88.83359 | 46.338332 | 751 | *Krascheninnikovia latens,Anabasis aphylla* | Temperate desert | 69.25 |
| 18 | 88.84036 | 46.140483 | 854 | *Seriphidium terrae-aibae,Stipa glareosa* | Temperate desert | 30.47 |
| 19 | 89.40452 | 46.465589 | 815 | *Seriphidium terrae-aibae,Stipa glareosa* | Temperate desert | 78.67 |
| 20 | 89.83616 | 45.957379 | 1042 | *Seriphidium terrae-aibae,Stipa glareosa* | Temperate desert | 27.82 |
| 21 | 89.93473 | 46.134884 | 910 | *Seriphidium terrae-aibae,Stipa glareosa* | Temperate desert | 65.37 |
| 22 | 89.56346 | 46.456057 | 865 | *Seriphidium terrae-aibae,Stipa glareosa* | Temperate desert | 89.75 |
| 23 | 89.37815 | 47.10364 | 823 | *Seriphidium terrae-aibae,Kochia prostrate, Stipa sareptana* | Temperate desert | 146.81 |
| 24 | 89.32071 | 47.018547 | 777 | *Seriphidium terrae-aibae,Kochia prostrate, Stipa sareptana* | Temperate desert | 108.03 |
| 25 | 89.07434 | 47.119275 | 824 | *Seriphidium terrae-aibae,Kochia prostrate, Stipa sareptana* | Temperate desert | 108.03 |
| 26 | 89.55073 | 46.919628 | 844 | *Seriphidium terrae-aibae,Kochia prostrate, Stipa sareptana* | Temperate desert | 162.33 |
| 27 | 89.56773 | 46.861914 | 919 | *Seriphidium terrae-aibae,Kochia prostrate, Stipa sareptana* | Temperate desert | 34.35 |
| 28 | 89.63992 | 46.751043 | 981 | *Seriphidium terrae-aibae,Kochia prostrate, Stipa sareptana* | Temperate desert | 92.52 |
| 29 | 89.84972 | 46.764503 | 1190 | *Seriphidium terrae-aibae,Kochia prostrate, Stipa sareptana* | Temperate desert | 54.29 |
| 30 | 89.74596 | 46.843294 | 958 | *Seriphidium terrae-aibae,Kochia prostrate, Stipa sareptana* | Temperate desert | 100.83 |
| 31 | 89.48725 | 47.061617 | 1067 | *Seriphidium terrae-aibae,Kochia prostrate, Stipa sareptana* | Temperate desert | 115.24 |
| 32 | 89.53292 | 46.15326 | 998 | *Seriphidium terrae-aibae,Kochia prostrate, Stipa sareptana* | Temperate desert | 76.45 |
| 33 | 89.52263 | 46.022846 | 995 | *Seriphidium kaschgaricum,Anabasis brevifolia, Stipa glareosa* | Temperate desert | 24.93 |
| 34 | 89.5334 | 46.22403 | 997 | *Seriphidium gracilescens,Kochia prostrate, Stipa glareosa* | Temperate desert | 74.79 |
| 35 | 89.49098 | 45.979375 | 979 | *Seriphidium gracilescens,Kochia prostrate, Stipa glareosa* | Temperate desert | 30.47 |
| 36 | 89.44138 | 45.758589 | 922 | *Anabasis salsa* | Temperate desert | 58.17 |
| 37 | 89.37512 | 45.801771 | 905 | *Seriphidium terrae-aibae,Anabasis salsa* | Temperate desert | 41.55 |
| 38 | 89.28247 | 45.836062 | 872 | *Seriphidium terrae-aibae,Anabasis salsa* | Temperate desert | 36.09 |
| 39 | 89.54574 | 45.720741 | 953 | *Climacoptera subcrassa* | Temperate desert | 33.83 |
| 40 | 89.50647 | 45.536094 | 993 | *Anabasis salsa* | Temperate desert | 36.09 |
| 41 | 89.47082 | 45.451669 | 1031 | *Seriphidium terrae-aibae,Stipa glareosa* | Temperate desert | 58.17 |
| 42 | 89.36914 | 45.3819 | 1042 | *Anabasis brevifolia,Stipa glareosa* | Temperate desert | 55.4 |
| 43 | 89.27349 | 45.90439 | 970 | *Seriphidium terrae-aibae,Stipa glareosa* | Temperate desert | 32.13 |
| 44 | 88.76753 | 45.6576 | 516 | *Seriphidium terrae-aibae,Stipa glareosa* | Temperate desert | 70.91 |
| 45 | 88.84992 | 45.695823 | 850 | *Krascheninnikovia latens,Artemisia desertorum* | Temperate desert | 83.1 |
| 46 | 88.84597 | 45.901017 | 800 | *Seriphidium terrae-aibae,Stipa glareosa* | Temperate desert | 36.57 |
| 47 | 89.522838 | 45.599727 | 961 | *Seriphidium terrae-aibae,Stipa glareosa* | Temperate desert | 96.95 |
| 48 | 89.444845 | 45.422011 | 1013 | *Seriphidium terrae-aibae,Stipa glareosa* | Temperate desert | 83.1 |
| 49 | 89.77127 | 47.122283 | 1146 | *Stipa capillata,Festuca ovina, Seriphidium terrae-aibae* | Temperate typical steppe | 93.98 |
| 50 | 89.80345 | 47.2323 | 1263 | *Stipa capillata,Festuca ovina, Seriphidium terrae-aibae* | Temperate typical steppe | 152.35 |
| 51 | 89.31333 | 47.3161 | 1400 | *Stipa capillata,Festuca ovina, Kochia prostrate* | Temperate typical steppe | 124.65 |
| 52 | 89.29287 | 47.2986 | 1362 | *Stipa capillata,Festuca ovina* | Temperate typical steppe | 167.54 |
| 53 | 89.7474 | 46.9734 | 1413 | *Seriphidium terrae-aibae,Stipa glareosa* | Temperate typical steppe | 93.98 |
| 54 | 88.47154 | 46.55346 | 651 | *Agrostis gigantea* | Lowland meadow | 307.69 |
| 55 | 88.77588 | 46.372236 | 700 | *Phragmites communis,Calamagrostis langsdorffii* | Lowland meadow | 488.42 |
| 56 | 89.11655 | 46.437741 | 752 | *Phragmites communis,Calamagrostis langsdorffii* | Lowland meadow | 266.67 |
| 57 | 89.20776 | 46.44034 | 761 | *Leymus multicaulis,Phragmites communis* | Lowland meadow | 287.72 |
| 58 | 89.3656 | 46.455895 | 788 | *Phragmites communis,Calamagrostis langsdorffii* | Lowland meadow | 407.02 |
| 59 | 89.88265 | 46.211869 | 857 | *Phragmites communis,Calamagrostis langsdorffii* | Lowland meadow | 421.05 |
| 60 | 89.45885 | 46.987167 | 729 | *Phragmites communis* | Lowland meadow | 401.4 |
| 61 | 89.69762 | 47.76405 | 2335 | *Poa alpine,Carex stenocarpa, Alchemilla pinguis* | Alpine meadow | 173.5 |
| 62 | 89.69333 | 47.764722 | 2600 | *Poa alpine,Carex stenocarpa, Alchemilla pinguis* | Alpine meadow | 143.38 |
| 63 | 89.5017 | 47.942869 | 2125 | *Carex stenocarpa,Polygonum viviparum* | Alpine meadow | 140.83 |
| 64 | 89.48135 | 48.010858 | 2213 | *Poa alpine,Carex stenocarpa, Alchemilla pinguis* | Alpine meadow | 158.98 |
| 65 | 89.49152 | 47.976864 | 2169 | *Poa alpine,Carex stenocarpa, Alchemilla pinguis* | Alpine meadow | 117.97 |
| 66 | 89.71113 | 47.593117 | 1617 | *Poa angustifolia,Alchemilla pinguis* | Mountain meadow | 169.74 |
| 67 | 89.7285 | 47.7058 | 1780 | *Poa angustifolia* | Mountain meadow | 165.79 |
| 68 | 89.74857 | 47.6772 | 1710 | *Poa angustifolia,Alchemilla pinguis* | Mountain meadow | 215.79 |
| 69 | 89.4295 | 47.403867 | 1702 | *Poa angustifolia,Alchemilla pinguis* | Mountain meadow | 228.07 |
| 70 | 89.37845 | 47.404783 | 1820 | *Poa angustifolia,Alchemilla pinguis* | Mountain meadow | 184.21 |
| 71 | 89.82101 | 47.6443 | 1882 | *Poa angustifolia,Alchemilla pinguis* | Mountain meadow | 249.12 |
| 72 | 89.41043 | 47.566131 | 2075 | *Poa angustifolia,Alchemilla pinguis* | Mountain meadow | 184.65 |
| 73 | 89.95782 | 47.911549 | 1502 | *Carex buekii,Poa angustifolia,  Potentilla chrysantha* | Mountain meadow | 59.21 |
| 74 | 89.39707 | 47.924257 | 1881 | *Carex buekii,Poa angustifolia,  Potentilla chrysantha* | Mountain meadow | 154.39 |
| 75 | 89.43808 | 47.937531 | 1924 | *Carex buekii,Poa angustifolia,  Potentilla chrysantha* | Mountain meadow | 153.51 |
| 76 | 89.44621 | 47.963503 | 1978 | *Carex buekii,Poa angustifolia,  Potentilla chrysantha* | Mountain meadow | 245.61 |
| 77 | 89.82402 | 47.685363 | 1526 | *Poa angustifolia,Alchemilla pinguis* | Mountain meadow | 104.39 |
| 78 | 89.10778 | 47.681793 | 1673 | *Poa angustifolia,Alchemilla pinguis* | Mountain meadow | 189.47 |
| 79 | 89.1646 | 47.643324 | 1753 | *Poa angustifolia,Alchemilla pinguis* | Mountain meadow | 142.54 |
| 80 | 89.47248 | 47.530562 | 2039 | *Poa angustifolia,Alchemilla pinguis* | Mountain meadow | 186.4 |
| 81 | 89.41833 | 47.554067 | 2027 | *Phragmites communis,Carex buekii* | Swamp | 110.53 |
| 82 | 89.7474 | 47.2022 | 1320 | *Phragmites communis,Carex buekii* | Swamp | 98.68 |
| 83 | 88.909 | 46.3989 | 810 | *Phragmites communis,Carex buekii* | Swamp | 87.06 |

**Table 2** NPP_A_ change in summer pasture induced by climate change and human activities in different townships (%)

|  | DR | KLBLG | KZLXLK | KET | TMK | TEH |
| --- | --- | --- | --- | --- | --- | --- |
| HI | 23.78 | 3.63 | 14.72 | 7.24 | 0.00 | 7.74 |
| BHI | 8.51 | 14.39 | 16.53 | 12.23 | 1.31 | 17.41 |
| HI+BHI | 32.29 | 18.01 | 31.25 | 19.47 | 1.31 | 25.15 |
| CI | 38.36 | 48.14 | 36.98 | 49.27 | 75.83 | 42.96 |
| BCI | 10.85 | 22.72 | 15.18 | 11.82 | 7.89 | 19.24 |
| CI+BCI | 49.21 | 70.85 | 52.15 | 61.09 | 83.72 | 62.20 |
| TI | 81.49 | 88.87 | 83.40 | 80.56 | 85.04 | 87.35 |
| HD | 16.00 | 9.39 | 14.89 | 17.95 | 14.96 | 12.22 |
| BHD | 1.17 | 1.20 | 0.60 | 0.70 | _ | 0.22 |
| HD+BHD | 17.17 | 10.59 | 15.49 | 18.65 | 14.96 | 12.44 |
| CD | 1.05 | 0.32 | 0.90 | 0.50 | _ | 0.14 |
| BCD | 0.29 | 0.22 | 0.21 | 0.29 | _ | 0.07 |
| CD+BCD | 1.34 | 0.54 | 1.11 | 0.79 | _ | 0.21 |
| TD | 18.51 | 11.13 | 16.60 | 19.44 | 14.96 | 12.65 |

DR: Dure town; KLBLG: Kalabulegen town; KZLXLK: Kezilexilike town; KET: Kuerte town; TMK: Tiemaike town; and TEH: Tuerhong town.

**Table 3** NPP_A_ change in spring and autumn pasture induced by climate change and human activities in different townships (%)

|  | DR | KLBLG | KLTK | KZLXLK | KET | TMK | TEH |
| --- | --- | --- | --- | --- | --- | --- | --- |
| HI | _ | _ | _ | _ | _ | _ | _ |
| BHI | 0.12 | 0.11 | 0.22 | 0.21 | 0.13 | 0.55 | 0.12 |
| HI+BHI | 0.12 | 0.11 | 0.22 | 0.21 | 0.13 | 0.55 | 0.12 |
| CI | 29.22 | 30.73 | 17.14 | 43.14 | 29.76 | 53.64 | 28.55 |
| BCI | 0.45 | 0.30 | 0.61 | 0.58 | 0.39 | 2.66 | 0.46 |
| CI+BCI | 29.66 | 31.03 | 17.76 | 43.72 | 30.15 | 56.29 | 29.01 |
| TI | 29.79 | 31.15 | 17.98 | 43.93 | 30.28 | 56.85 | 29.13 |
| HD | 70.21 | 68.85 | 82.02 | 56.07 | 69.72 | 43.15 | 70.87 |
| BHD | _ | _ | _ | _ | _ | _ | _ |
| HD+BHD | 70.21 | 68.85 | 82.02 | 56.07 | 69.72 | 43.15 | 70.87 |
| CD | _ | _ | _ | _ | _ | _ | _ |
| BCD | _ | _ | _ | _ | _ | _ | _ |
| CD+BCD | _ | _ | _ | _ | _ | _ | _ |
| TD | 70.21 | 68.85 | 82.02 | 56.07 | 69.72 | 43.15 | 70.87 |

DR: Dure town; KLBLG: Kalabulegen town; KZLXLK: Kezilexilike town; KET: Kuerte town; TMK: Tiemaike town; and TEH: Tuerhong town.

**Table 4 NPP_A_** change in winter pasture induced by climate change and human activities in different townships (%)

|  | DR | KLBLG | KLTK | KZLXLK | KET | TMK | TEH |
| --- | --- | --- | --- | --- | --- | --- | --- |
| HI | _ | _ | _ | _ | _ | _ | 2.56 |
| BHI | 0.13 | 0.11 | 0.38 | 0.02 | 0.04 | 0.10 | 0.71 |
| HI+BHI | 0.13 | 0.11 | 0.38 | 0.02 | 0.04 | 0.10 | 3.27 |
| CI | 17.94 | 8.53 | 13.29 | 38.98 | 20.62 | 11.21 | 22.25 |
| BCI | 0.22 | 0.15 | 0.88 | 0.16 | 0.20 | 0.03 | 0.81 |
| CI+BCI | 18.17 | 8.68 | 14.16 | 39.14 | 20.83 | 11.23 | 23.06 |
| TI | 18.29 | 8.79 | 14.55 | 39.16 | 20.87 | 11.34 | 26.33 |
| HD | 81.71 | 91.21 | 85.45 | 60.84 | 79.13 | 88.66 | 70.24 |
| BHD | _ | _ | _ | _ | _ | _ | 0.63 |
| HD+BHD | 81.71 | 91.21 | 85.45 | 60.84 | 79.13 | 88.66 | 70.87 |
| CD | _ | _ | _ | _ | _ | _ | 2.15 |
| BCD | _ | _ | _ | _ | _ | _ | 0.65 |
| CD+BCD | _ | _ | _ | _ | _ | _ | 2.80 |
| TD | 81.71 | 91.21 | 85.45 | 60.84 | 79.13 | 88.66 | 73.67 |

DR: Dure town; KLBLG: Kalabulegen town; KZLXLK: Kezilexilike town; KET: Kuerte town; TMK: Tiemaike town; and TEH: Tuerhong town.
